# Understanding and Controlling the Crystallization Process in Reconfigurable Plasmonic Superlattices

**DOI:** 10.1021/acsnano.0c09746

**Published:** 2021-02-23

**Authors:** Maciej Bagiński, Adrián Pedrazo-Tardajos, Thomas Altantzis, Martyna Tupikowska, Andreas Vetter, Ewelina Tomczyk, Radius N.S. Suryadharma, Mateusz Pawlak, Aneta Andruszkiewicz, Ewa Górecka, Damian Pociecha, Carsten Rockstuhl, Sara Bals, Wiktor Lewandowski

**Affiliations:** † Faculty of Chemistry, University of Warsaw, 1 Pasteura St., 02-093 Warsaw, Poland; ‡ Electron Microscopy for Materials Research, University of Antwerp, Groenenborgerlaan, 171, 2020 Antwerp, Belgium; § Institute of Theoretical Solid State Physics, Karlsruhe Institute of Technology, 76131 Karlsruhe, Germany; ∥ Department of Chemistry, Uppsala Universitet, Lägerhyddsvägen 1, 751 20 Uppsala, Sweden; ⊥ Institute of Nanotechnology, Karlsruhe Institute of Technology, 76021 Karlsruhe, Germany

**Keywords:** TEM tomography, *in situ* TEM, liquid crystals, plasmonics, dynamic assembly, supramolecular self-assembly, cooperative interactions

## Abstract

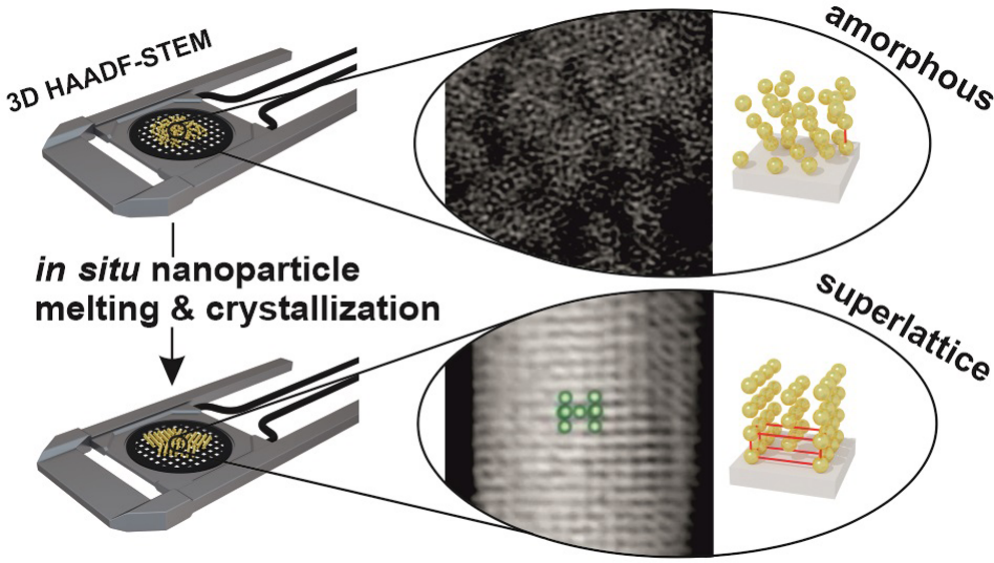

The crystallization of nanomaterials is a primary source of solid-state, photonic structures. Thus, a detailed understanding of this process is of paramount importance for the successful application of photonic nanomaterials in emerging optoelectronic technologies. While colloidal crystallization has been thoroughly studied, for example, with advanced *in situ* electron microscopy methods, the noncolloidal crystallization (freezing) of nanoparticles (NPs) remains so far unexplored. To fill this gap, in this work, we present proof-of-principle experiments decoding a crystallization of reconfigurable assemblies of NPs at a solid state. The chosen material corresponds to an excellent testing bed, as it enables both *in situ* and *ex situ* investigation using X-ray diffraction (XRD), transmission electron microscopy (TEM), high-angle annular dark-field scanning transmission electron microscopy (HAADF-STEM), atomic force microscopy (AFM), and optical spectroscopy in visible and ultraviolet range (UV–vis) techniques. In particular, ensemble measurements with small-angle XRD highlighted the dependence of the correlation length in the NPs assemblies on the number of heating/cooling cycles and the rate of cooling. *Ex situ* TEM imaging further supported these results by revealing a dependence of domain size and structure on the sample preparation route and by showing we can control the domain size over 2 orders of magnitude. The application of HAADF-STEM tomography, combined with *in situ* thermal control, provided three-dimensional single-particle level information on the positional order evolution within assemblies. This combination of real and reciprocal space provides insightful information on the anisotropic, reversibly reconfigurable assemblies of NPs. TEM measurements also highlighted the importance of interfaces in the polydomain structure of nanoparticle solids, allowing us to understand experimentally observed differences in UV–vis extinction spectra of the differently prepared crystallites. Overall, the obtained results show that the combination of *in situ* heating HAADF-STEM tomography with XRD and *ex situ* TEM techniques is a powerful approach to study nanoparticle freezing processes and to reveal the crucial impact of disorder in the solid-state aggregates of NPs on their plasmonic properties.

## Introduction

Ordered nanoparticle solids (superlattices (SLs)) are very interesting materials with an overarching importance for future photonic,^1−4^ metamaterial,^5−7^ electronic,^8,9^ optical,^10^ and energy-related applications.^11^ This interest can be attributed to their resonant, transport, and mechanical functionalities. Precise control of these properties is required for an efficient exploitation of NP SLs; thus, a lot of effort was put into investigating the influence of particle type and spatial distribution of particles within the solid. Recently, it was recognized that a successful integration of periodic assemblies of NPs into optoelectronic devices^12^ is also critically dependent on the macroscopic uniformity,^13^ (poly)domain structure of these artificial solids,^14,15^ and their defects.^16^ For instance, cracks present in a given superlattice^17^ can lead to unexpected optical properties, for example, in Ag NPs solids.^18^ Therefore, understanding the NP SL formation process at the combined single particle, single domain, and macroscopic levels is essential to fully control the structure and properties of the obtained materials.

The two main approaches for NP SL formation are (directed) colloidal crystallization^19−21^ and neat-state crystallization (freezing).^22,23^ The former has been widely explored, for example, by UV/vis, X-ray diffraction (XRD),^24^ transmission electron microscopy (TEM), and X-ray cross-correlation^25^ analyses. Lately, *in situ* TEM investigations of NP colloidal crystallization at the single-particle and the ensemble levels were enabled^26,27^ using liquid-phase measurements. Particularly, these measurements allowed for a direct observation of the long-range order emergence.^27^ However, in the case of a noncolloidal crystallization of reconfigurable NP solids,^28−31^ the available toolbox, regarding TEM analyses, is limited to *ex situ* techniques. Furthermore, these investigations are often limited to two-dimensional (2D) projection measurements, and the information provided is insufficient to monitor and study the heat-induced three-dimensional (3D) morphological changes and deformation of highly ordered and complex structures, such as superlattices.^32^ Indeed, currently, *in situ* TEM studies of such ensembles “during formation”^33^ or during a 3D analysis of NP solids have been rarely reported, mainly due to equipment- and material-dependent limitations. Nowadays, dedicated tomography TEM holders exist in which tilting to high angles and heating functionalities are combined. These holders, however scarce, enable researchers to perform *in situ* thermal studies in 3D.^34−36^ In this manner, it becomes possible to study heat-induced 3D structural and compositional changes of complex structures at different stages of thermal treatment.^37−39^ Nevertheless, the investigation of reversibly reconfigurable systems is problematic due to material-related issues^40^ such as the relatively low stability of organic materials (*e.g*., surface ligands of NPs) when exposed to the electron beam.

One of the most promising reconfigurable nanomaterials, from the applicative point of view, is NPs covered with liquid-crystalline ligands (LC NPs). Driven by the reorientation of LC ligands around nanocrystal cores in the neat state, these NPs tend to form ordered solids^41−45^ in a fashion similar to that of purely organic, thermotropic LC materials. Hierarchical, nonclose-packed, long-range ordered structure, and (remote) stimuli responsiveness^46^ are typical features of LC-covered NP solids. Such assemblies of NPs up to 11 nm in diameter^47^ were recently exploited for the construction of adaptive plasmonic and photonic nanomaterials,^48,49^ as well as (switchable) metamaterials,^6^ making them interesting for detailed structural studies of the noncolloidal crystallization process.^50^

Here, we study the emergence of order in NP solids at the mesoscale level *in situ*, focusing on the structure of individual domains of NPs. For this purpose, we used liquid-crystalline NPs that can be reversibly rearranged between different phases by varying the substrate temperature. Using small-angle X-ray diffraction (SAXRD) and UV–vis spectroscopic measurements we examined the collective behavior of NPs at the bulk level, developing an understanding of their averaged dynamic structure and properties, determined by the crystallization conditions. TEM measurements, including tomography studies of *in situ* thermally annealed samples, allowed to develop an understanding of the superlattice structure at different hierarchical levels. By complementing these results with the UV–vis absorption modeling we were able to correlate differences in the plasmonic properties of NPs solids to the decreased positional order of NPs at crystallite (NP domains) boundaries.

## Results/Discussion

### Au@L Nanoparticles Design and Synthesis

**Figure 1:**
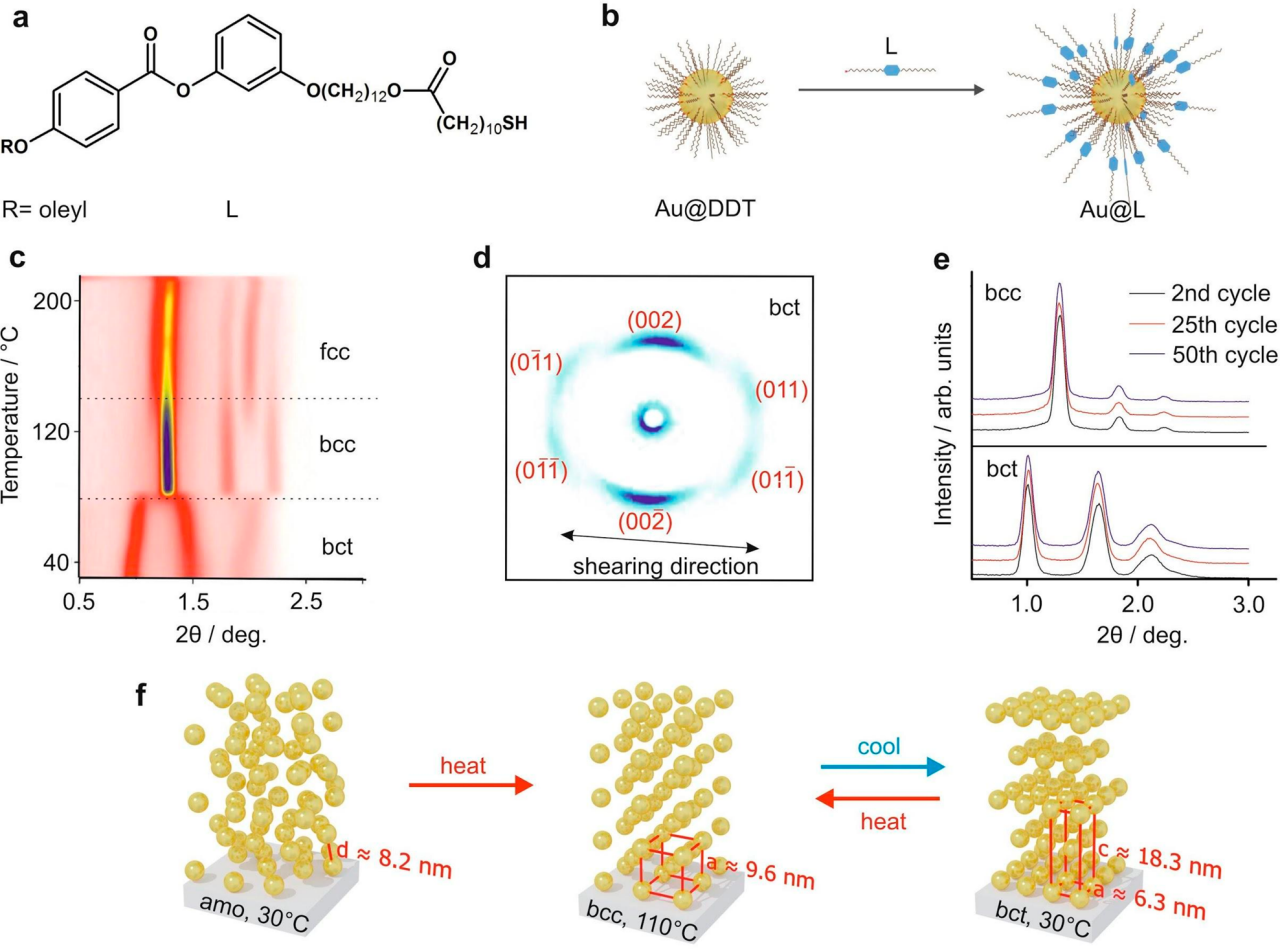
Design and structural investigations of Au@L material. (a) Molecular structure of a promesogenic ligand L used for nanoparticle surface modification. (b) A scheme of the ligand exchange reaction. (c) Temperature evolution of an SAXRD pattern obtained by the heating of an annealed sample. (d) Diffractogram obtained at 30 °C for a mechanically sheared sample. (e) SAXRD diffractograms collected at 30 °C (body-centered tetragonal phase, bct) and 110 °C (body-centered cubic phase, bcc); vertically shifted diffractograms from the 2nd, 25th, and 50th heating/cooling cycles are shown for clarity. (f) Schematic structure of Au@L material directly after being dropcasted (amorphous phase, amo), after being heated to 110 °C (bcc), and after being cooled at 30 °C (bct); organic ligands are not shown for clarity.

To obtain long-range-ordered, thermoswitchable assemblies of NPs we prepared NPs coated with a liquid-crystal-like (promesogenic) surface ligand, synthesized following a previously developed route.^51^ The molecular architecture of the promesogenic ligand (L, Figure 1a) was based on a bicyclic central aromatic core equipped with two alkyl chains. The first enabled docking of L at the nanoparticle surface (via a mercapto moiety) and ligand reorientation around the nanocrystal core. The second, an oleyl chain, endowed ligands with a degree of fluidity. Overall, L was designed to support an efficient formation of anisotropic, thermally switchable assemblies of NPs as previously shown.^51^

As the platform for building LC NPs, we decided to use hydrophobic, dodecanethiol-coated 4.1 nm diameter spherical Au NPs (Au@DDT, TEM image, and histogram of size distribution are given in Figure S1) prepared using a modified method as described in the literature.^52^ The size of the NPs, which is comparable to the length of L, and their low size dispersity (below 10%) favor the formation of long-range ordered assemblies of NPs. Importantly, the examined NPs exhibit a clear plasmonic band (centered at ca. 520 nm in a toluene dispersion, Figure S2), which enables a probing correlation between the structure and optical properties of NP assemblies.

To obtain the final material (Au@L) we introduced L to the surface of Au@DDT NPs using a solution-phase ligand-exchange procedure^53^ (Figure 1b). Confirmation of the presence of L ligands in Au@L was provided by a thermogravimetric analysis (TGA, Figure S3a) and NMR studies (Figure S3b,c; a detailed description of these measurement results is given in the Experimental Section). Quantitative information on the organic shell composition was elucidated from the TGA data; ∼35% of dodecanethiol molecules were exchanged to L ligands.

### Reconfigurable Assembly of Au@L Nanoparticles

First, we used SAXRD to analyze *in situ* the thermoresponsive assembly of the Au@L material in a condensed state. For this purpose, a Au@L dispersion was dropcasted onto a Kapton tape and heat annealed to achieve a thermodynamically favored arrangement of nanoparticles (30–120–30 °C heating/cooling cycle, cooling rate 3 °C/min). The evolution of the SAXRD pattern during heating revealed the formation of three distinct ordered phases (Figure 1c). Below 80 °C, the SAXRD pattern can be fitted assuming a 3D body-centered tetragonal phase (bct, Figure S4a, Table S1) with unit cell dimensions changing from *a* ≈ 6.3 nm and *c* ≈ 18.3 nm at 30 °C to *a* ≈ 6.6 and *c* ≈ 17.2 nm at 70 °C. Although samples prepared by dropcasting typically have a random, polydomain structure, it was possible to induce a partial alignment of the domains by a mechanical shearing of the material. The XRD pattern of such an aligned sample in the bct phase revealed two sets of discrete peaks in directions perpendicular and oblique to the shearing direction (Figure 1d). At 80 °C, a phase transition to another long-range-ordered phase was observed. The corresponding XRD pattern was fitted with high accuracy assuming a body-centered cubic symmetry (bcc) with a lattice parameter of *a* ≈ 9.6 nm at 110 °C (Figure S4b). Above 130 °C, SAXRD measurements revealed another reconfiguration of the NPs’ spatial arrangement to a face-centered cubic phase (fcc) with a lattice parameter of *a* ≈ 12.3 nm at 140 °C (Figure S4c). The fcc phase was stable up to 215 °C, while further heating resulted in a rapid decomposition of the sample, as evidenced by the growing intensity of X-rays scattered around the beam stopper. SAXRD measurements during cooling revealed the same phase sequence as observed while heating (Figure S5) and showed a relatively low phase-transition temperature hysteresis, which is typical for LC materials.^54^ We also confirmed that Au@L can be reversibly reconfigured between bct and bcc phases (between 30 and 110 °C) several times, as evidenced by the reproducibility of XRD patterns (Figure 1e). Taking into account that directly after dropcasting the nonannealed assembly is amorphous (amo), with an average interparticle distance of 8.2 nm (Figure S6a,b), we elucidated the evolution of the NPs’ solid structure *versus* thermal treatment of the sample (Figure 1f).

### Structural Impact of Crystallization Conditions

**Figure 2:**
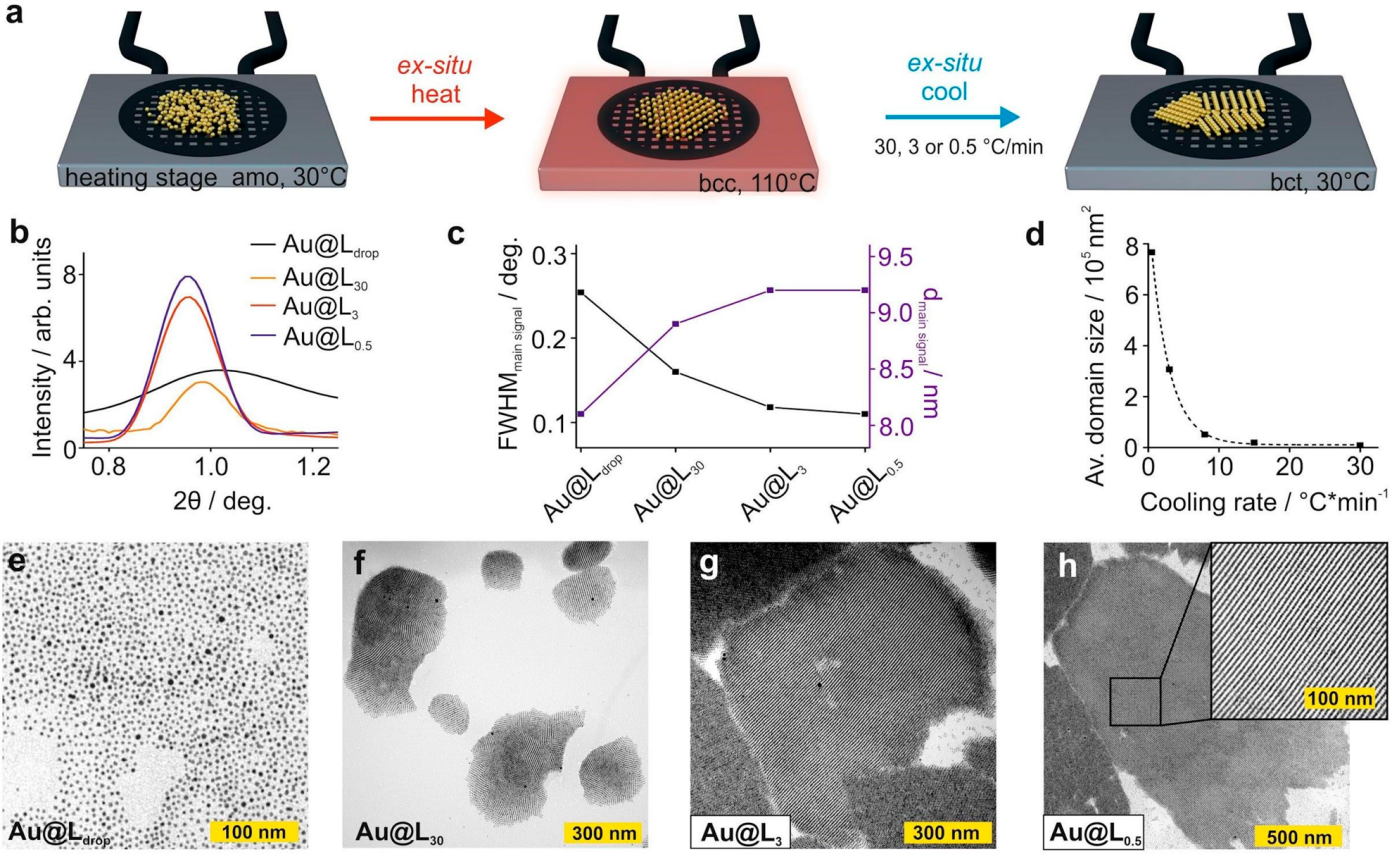
*Ex situ* investigation of the crystallization conditions effects on Au@L material structure, including directly dropcasted (Au@L_drop_) and heat-annealed samples (Au@L_30_, Au@L_3_, and Au@L_0.5_, subscripts indicate cooling rates). (a) Scheme of *ex situ* sample preparation for the TEM and SAXRD measurements. (b) Comparison of the main peak region of one-dimensional diffractograms obtained for the samples. (c) Comparison of the position and fwhm of the main XRD peaks shown in panel (b); the solid lines serve as a guide. (d) Mean areas of nanoparticle domains for samples crystallized at different cooling rates; the dashed line is for guidance. (e–h) TEM images of the Au@L samples crystallized at different cooling rates (insets in the bottom left corner indicate preparation conditions).

After determining the reconfigurable nature of Au@L NPs, we decided to study the bct phase formation in detail. We were particularly interested in assessing the impact of the thermal annealing conditions on the structure of formed NPs solid. Therefore, we focused on bulk-scale XRD measurements, as an evaluation of the position and full width at half maxima (fwhm) of the main XRD peak provides information on the periodicity and positional correlation length within the probed material, respectively. Complementary information at the single-particle level was accessed using *ex situ* TEM measurements (Figure 2a).

One of the parameters that can influence the domain size of soft materials is the number of annealing cycles. We therefore aimed at determining the minimum number of heating/cooling cycles after which we do not see an increase of positional correlation lengths in the Au@L bct phase. For this purpose, we analyzed the main XRD peak from diffractograms collected directly after dropcasting (Au@L_drop_) as well as after the sample was heated to 120 °C (ratio 30 °C/min) and cooled to 30 °C (ratio 3 °C/min) one, two, or three times. As mentioned above, the XRD diffractogram of the dropcasted sample revealed a single, broad (fwhm ∼0.262°) signal, indicating only a short-range ordered character of the assembly. After the first heat annealing cycle the fwhm of the main, (002), XRD signal of bct phase was much narrower (∼0.132°, Figure S6a), indicating a long-range-ordered character of the sample. The second cycle resulted in a further decrease of the fwhm value to ∼0.114°, while consecutive cycles had a negligible influence on the XRD signal width (Figure S6b). Thus, we can conclude that, already after the second heating/cooling cycle, a long correlation length of the Au@L NPs positions is achieved.

To complement the above-discussed bulk-scale measurements with single-particle-level information we prepared samples exposed to different numbers of annealing cycles for conventional (*ex situ*) TEM imaging. For the dropcasted Au@L_drop_ sample, the dominant structure was comprised of isotropically arranged NPs (Figure S6c), as previously deduced from XRD studies. In the case of heat-annealed samples, domains of NPs arranged into vertically oriented layers were imaged, with an interlayer distance of ∼8.2 nm, corresponding well to the XRD-derived *c*/2 dimension (∼8.6 nm) of the bct unit cell. In this manner, we could use these images to estimate the impact of the number of heating/cooling cycles on the size of obtained bct domains (Figure S6c–e). After the first cycle, we observed domains of anisotropically ordered NPs with a mean area of ∼2.5 × 10^4^ nm^2^ (Figure S6d and Figure S7a–c). An increase of the mean domain size (up to ∼3 × 10^5^ nm^2^, Figure S6e and Figure S7d–f) was achieved for the sample that underwent the heating/cooling cycle twice. In analogy to the XRD results, a further increase in the number of annealing cycles did not result in an additional growth of the size of the domain. To confirm that the height of the formed domains is uniform, we prepared a Au@L sample on a TEM grid (heat-annealed, cooling rate 3 °C/min) which was subject to atomic force microscopy (AFM) measurements. Domains with morphologies similar to those found by TEM were also observed by AFM (Figure S8a,b). These measurements confirmed a uniform height of the domains in the range of 55–60 nm (Figure S8c), corresponding to six to seven layers of NPs. We conclude that both XRD and TEM measurements indicated that the highest correlation lengths can be achieved already after two heating/cooling cycles. We, therefore, followed this approach for the sample preparation in the remainder of this work.

The cooling rate is another parameter that can determine the crystallization path of soft materials.^55,56^ To investigate its influence on Au@L crystallization and to determine the optimal cooling rate, we prepared samples using 30 (Au@L_30_), 3 (Au@L_3_), and 0.5 (Au@L_0.5_) °C/min cooling rates. In all cases, samples were heated at a rate of 30 °C/min, since the heating kinetics does not affect the quality of the assemblies. XRD measurements did not reveal a cooling-rate-dependent polymorphism—in all cases, a bct phase was formed. Moreover, we confirmed that the dimension of the bct unit cell is also independent of the cooling rate, as we observed only slight (3%) changes of the main XRD peak position (Figure 2b,c). However, a clear difference of fwhm values of the main diffraction peak was detected; the fwhm decreased with a reducing cooling rate: 0.152 (30 °C/min), 0.114 (3 °C/min), and 0.108 (0.5 °C/min, Figure 2b,c) degrees. Considering the polydispersity of nanoparticles (which cause small fluctuations of the interparticle distances) and the finite size of crystallites in the bct phase, it can be concluded that the saturated fwhm value of 0.108° was close to the equipment-related fwhm limit, which is ∼0.08° (defined mainly by beam divergence and detector resolution), indicating the long-range character of the sample. The TEM imaging of samples prepared using analogous cooling rates revealed domains of anisotropically ordered NPs and allowed us to confirm that the *c*-dimension of the unit cell was independent of the varied parameter, in qualitative agreement with the XRD-derived information. Conversely, the areas of uniform domains varied from 7 × 10^3^ nm^2^, via 3 × 10^5^ nm^2^, to 8 × 10^5^ nm^2^ for cooling rates of 30, via 3, to 0.5 °C/min, respectively (Figure 2e–h). With additional measurement points (Figure 2d; corresponding TEM images are given in Figure S9) an exponential growth of the average domain’s surface area with decreasing the cooling rate was revealed.

### *In Situ* Investigation of the Impact of Crystallization Conditions

**Figure 3:**
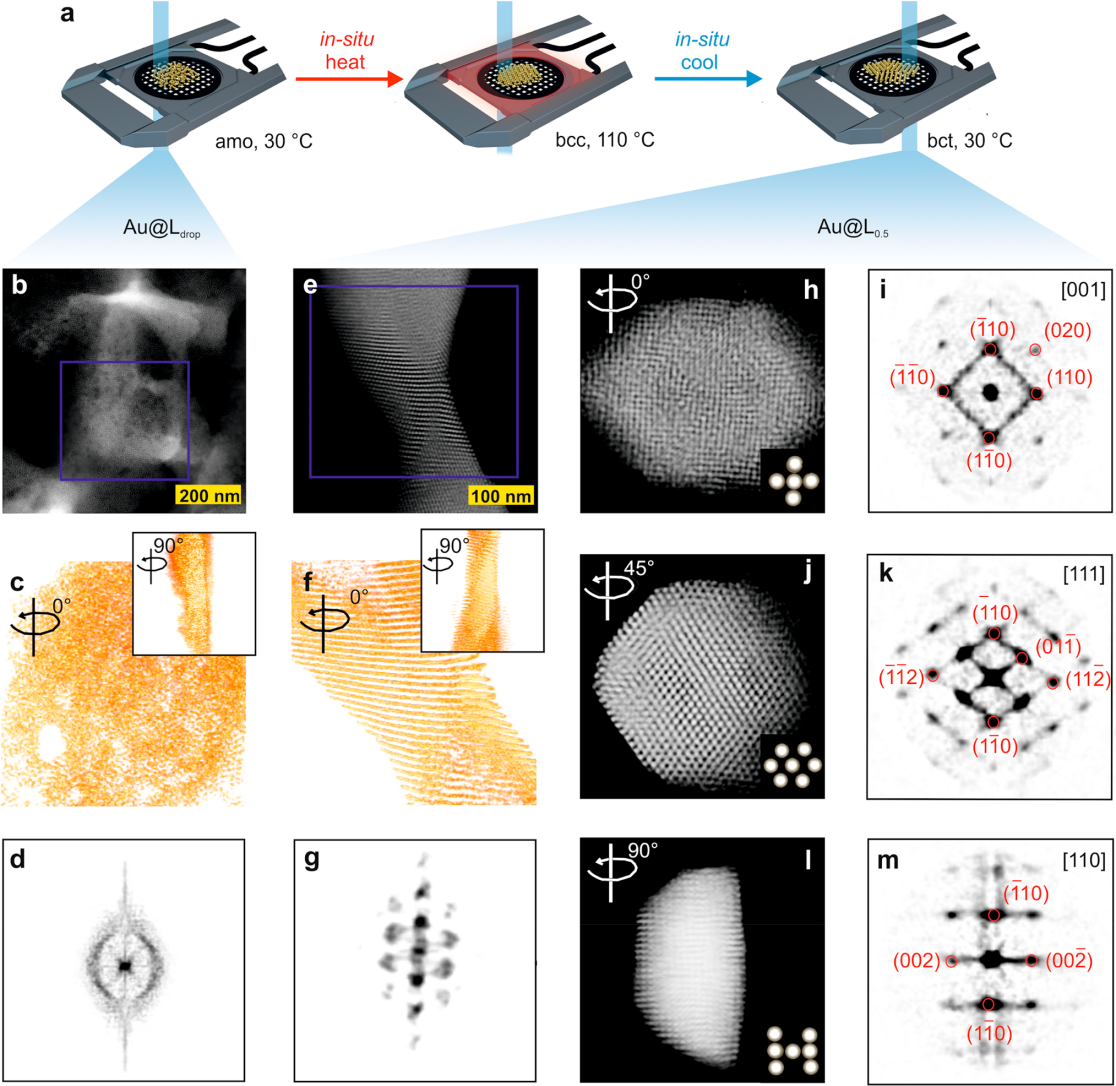
*In situ* investigation of the crystallization conditions effects on the Au@L material structure. (a) Schematic illustration of the *in situ* tomography measurements. (b) HAADF-STEM image of a directly dropcasted sample (Au@L_drop_). (c) 3D reconstructed volume of the region indicated by the blue square in panel (b). (inset) The structure rotated by 90°. (d) 3D-FFT projection image of the reconstruction shown in panel (c). (e) HAADF-STEM image of a heat-annealed sample (0.5 °C/min cooling rate, Au@L_0.5_). (f) 3D reconstructed volume of the region indicated by the blue square in panel (e). (inset) The structure rotated by 90°. (g) 3D-FFT projection image of the reconstruction shown in panel (f). (h, j, l) Projection images at different angles from the tomographic reconstruction acquired from a different region of the Au@L_0.5_ sample. The assembly in (j, l) is rotated 45° and 90°, respectively (rotation angle is given in the upper left corner). The insets at the bottom right corner show the corresponding unit cells. (i, k, m) FFT images acquired from the tomography shown in panels (h, j, l), respectively.

By exploiting recent developments in the field of the *in situ* 3D characterization of nanomaterials,^26,27^ we performed an *in situ* tomography experiment for Au@L samples. We hereby collected a tomographic series after evaporation-driven crystallization (Au@L_drop_) and crystallization by phase transition (Au@L_0.5_, Figure 3a).

The acquired high angle annular dark field scanning TEM (HAADF-STEM) projection images, 3D reconstructions, and 3D fast Fourier transforms (FFTs) (Figure 3, the first and the second column) evidence a difference in the symmetry of NP assemblies depending on the crystallization conditions. Namely, directly after the dropcasting no long-range positional ordering of NPs is visible in the HAADF-STEM image of an ensemble of NPs (Figure 3b). After collecting a tomography series in the HAADF-STEM mode, we obtained a 3D reconstruction of an ensemble (Figure 3c, Movie S1) comprising ∼10^4^ NPs with ∼300 nm diameter and ∼40 nm thickness, which further confirms the lack of anisotropic ordering of NPs. This information is strengthened by the corresponding 3D-FFT projection image (Figure 3d), which shows a single, broad signal corresponding to an amorphous phase.

Without removing the sample from the microscope, the material was heat-annealed with a cooling speed of 0.5 °C/min, which corresponds to the optimal conditions for a Au@L sample preparation (namely, the ones used to prepare Au@L_0.5_ material). Note that a direct visualization of the same area did not allow us to observe the rearrangement of NPs. This finding is likely related to damaged organic ligands or the presence of carbon contamination affecting the electron beam on the sample. Indeed, it has already been shown in the past that *in situ* electron beam irradiation can cause a high thermal stability of NPs assemblies, after being embedded in a carbon matrix. The primary carbon source of this carbon matrix is probably the coating agent on the nanocrystals, resulting in the production of a homogeneous carbon matrix.^57,58^ However, in all other parts of the grid, regions showing long-range-ordered, layered-type assemblies of NPs were visible in the HAADF-STEM image (Figure 3e). Importantly, these assemblies were of a similar thickness as the dropcasted structures, indicating that they were formed directly from the dropcasted material by thermal annealing, although the process was taking place at a very low pressure (∼10^–6^–10^–7^ Pa). 3D tomographic reconstruction (Figure 3f, Movie S3) and discrete diffraction spots observed in the corresponding projected 3D-FFT image (Figure 3g) confirmed that NPs exhibit 3D long-range order.

To study whether the *in situ* annealing method has any effect on the symmetry of the Au@L_0.5_ material, we focused on the detailed analysis of the arrangement of NPs in a single domain (Figure 3h–m). In the tomographic reconstruction, a single domain was found, separated from a multidomain region, and oriented along the [001] plane (Figure 3h, inset in the bottom right corner indicates the unit cell orientation, Movies S4 and S5), which we define as 0° turn. The corresponding slice from the 3D-FFT (Figure 3i) revealed a series of discrete Bragg reflections that can be indexed according to the (110) and (020) families, characteristic of the bct phase-oriented along the [001] plane. By rotating the domain by 45° and 90° we noted that the corresponding FFT patterns show diffractograms characteristic of [111] and [110] orientations of a bct structure. For a quantitative comparison, we calculated the inverse FFT to obtain the unit cell (Figure S10) in real space; a mean size of *c* ≈ 10.0–13.0 nm and *a* ≈ 6.3–6.7 nm were found. The difference along the *c*-axis, calculated based on XRD measurements (*c* ≈ 18.3 nm), 2D HAADF-STEM images (*c* ≈ 14.8 nm, Figure S11), and the 3D tomographic reconstruction (*c* ≈ 10.0–13.0 nm), can be explained based on the electron-beam-induced contraction of the material. The latter required collecting a time series of HAADF-STEM images, leading to a pronounced contraction effect (Figure S12, Movie S6). It is worth highlighting that a contraction did not change the symmetry exhibited by NPs, while affecting only the unit cell size.

### Functional Impact of Crystallization Conditions

The final goal of our research was to answer the question of how the variation of the crystallization conditions translates to controlling the optical properties of the material. In particular, we focused on the analysis of the plasmonic band maxima position, which is highly sensitive to changes in the spatial distribution of nanoparticles.^49^ We thus prepared a series of thin films of Au@L_drop_, Au@L_3_, and Au@L_0.5_ on a glass substrate and performed UV–vis measurements.

**Figure 4:**
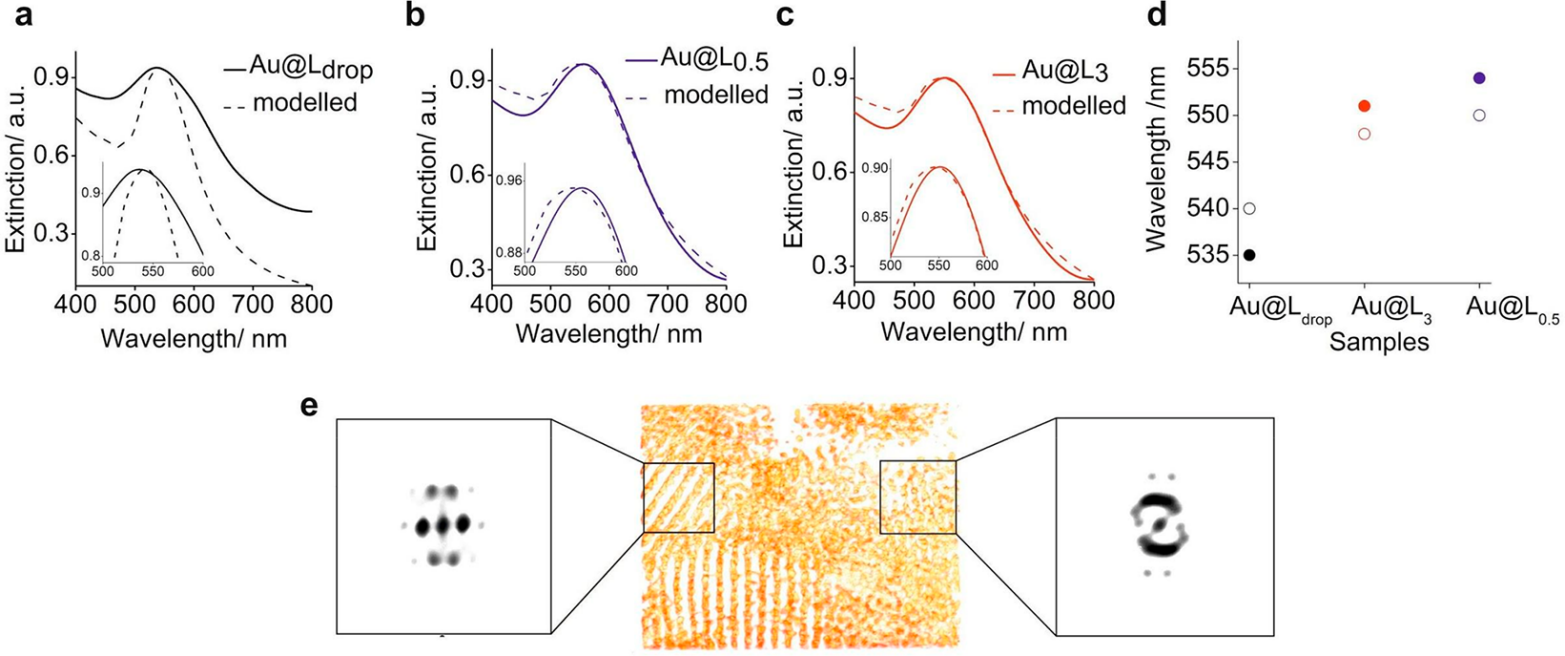
Optical and tomographic characterization of the Au@L material. Comparison of experimental and simulated extinction spectra for Au@L NPs (inset shows a magnified region of the plasmonic bands’ maxima) for (a) Au@L_drop_, (b) Au@L_0.5_, and (c) Au@L_3_ samples. (d) Comparison between modeled (empty circles) and experimental (filled circles) spectral positions of the surface plasmon resonance maxima for samples presented in panels (a–c). (e) Tomographic reconstruction of the Au@L_3_ sample and 3D-FFTs—from the center (on the left) and the border (on the right) regions of a single domain. The 3D-FFT from the specific regions allows for a qualitative analysis of the order in those regions. Region 2 is less ordered, as the presence of the amorphous ring in the 3D-FFT indicates that contribution.

A clear difference in the position of plasmonic band maxima was evidenced between Au@L_drop_ (535 nm, Figure 4a) and Au@L_0.5_ (554 nm, Figure 4b) samples. We know from SAXRD and TEM measurements that heat annealing induces reorganization from the amorphous to the bct phase. Thus, the observed plasmonic band shift can be well-explained by different distances between NPs in amorphous and bct phases. In the case of Au@L_3_, the plasmonic band maximum is slightly blueshifted (551 nm, Figure 4c) in comparison to that of the Au@L_0.5_ sample, which should originate from the structural difference between samples cooled at different rates.

To fully explain the observed differences in plasmonic resonances, we modeled the extinction of Au@L assemblies with structural parameters derived from the SAXRD measurements. The modeling was first performed assuming an “ideal” arrangement of Au NPs in different phases. The nondispersive refractive index of the medium surrounding the NPs of 1.5 was chosen based on the best fit for all samples. For modeling the Au NPs, we used established permittivity data^59^ in combination with a size correction for the imaginary part. Details of the modeling procedure can be found in the Experimental Section. In a nutshell, the method consists of solving Maxwell’s equations with a dedicated multiple scattering formalism that exploits both the spherical shape of the inclusions and their periodic arrangement.

The spectral positions of the plasmonic bands of Au@L_drop_ and Au@L_0.5_ samples are in a very good agreement with the model predictions (Figure 4a,b, respectively, a comparison of modeled and experimental plasmonics band maxima positions is given in Figure 4d and Table S2). For the Au@L_0.5_, fwhm values of the plasmonic bands are also perfectly reproduced by the model. This effect is also observed for the bcc structure (Figure S13), and thus it seems to be characteristic of long-range-ordered samples. Deviation of the modeled fwhm is observed for the short-range ordered Au@L_drop_ sample.

However, the experimentally observed difference in the spectral response of Au@L_3_*versus* Au@L_0.5_ samples is not accounted for in the used model. Therefore, we considered structural disorder, that is, a random displacement of the NPs about their lattice position, to reproduce the spectral response of Au@L_3_. In this manner, numerical modeling revealed that this influence on the optical response is negligible and hence does not account for the experimental findings either. Consequently, it appears that the optical response is not uniquely related to a single phase only. Instead, it is rather dominated by a bct phase but accompanied by a different structure. Analysis of the TEM images of a Au@L_3_ sample allowed us to note structural differences between internal and outer parts of the formed domains (Figure S14), which underpins this understanding; that is, NPs adopt the bct phase in the inner part of the domain, while an isotropic distribution of NPs is observed in the outer parts of domains. To further confirm these results, we performed electron tomography of a chosen Au@L_3_ domain (Figure 4e, Movie S2). FFT analysis of the inner part of the domain revealed discrete signals, characteristic of a well-developed bct structure, while the FFT image obtained from the outer part comprised both discrete and continuous signals coming from an amorphous phase.

To model the optical response of Au@L_3_, we, therefore, considered the optical response from individual bct and amorphous domains that are assumed to be spatially separated. Spatial separation of the domains is not a crucial issue because anyhow the plasmonic response is dominated by the interaction with the nearest neighbor in the lattice. When illuminating the sample in the UV–vis spectrometer, the optical response turns out to be an average of the plasmonic behavior for the individual domains. In a good approximation, the extinction is thus given by the average of the amo and bct spectra, weighted by the relative area coverage, calculated based on the experimentally observed relative shift between Au@L_0.5_ and Au@L_3_, in comparison to the shift between Au@L_0.5_ and Au@L_drop_ samples. We obtain an excellent agreement between the theoretical modeling and experimental measurements for Au@L_3_ (as shown in Figure 4c), assuming a relative bct coverage of 84%.

## Conclusions

In summary, by combining data from different techniques (SAXRD, *ex situ* TEM, *in situ* TEM), we were able to study the crystallization process of thermally switchable NPs assemblies in the solid state. These methods provided us with detailed information on the structure of the material both at the bulk scale and at the level of individual nanoparticles. At the bulk scale, we determined the material’s structure using SAXRD derived data: diffraction patterns enabled us to retrieve the symmetry of NPs domains, while the fwhm of a chosen XRD peak allowed us to assess the degree of alignment of nanoparticles within the domains. The size of the emerging domains was determined by a combination of TEM and AFM measurements. The structural analysis was further corroborated by single-particle precision *in situ* TEM measurements, which provided an insight into the 3D arrangement of NPs for various crystallization conditions. Importantly, the gathered information guided the optimization of the crystallization process. Namely, by changing the number of thermal annealing cycles and/or cooling rate we were able to obtain up to micron-size, long-range-ordered domains of NPs.

We also confirmed that the observed structural differences translated to the variable, bulk spectroscopic properties of the thin film of NPs. By combining *in situ* and *ex situ* TEM measurements with the UV–vis modeling we highlight the crucial role of the arrangement of NPs around domain boundaries on the plasmonic band maxima position, which is an important step toward understanding details of the crystallization process of stimuli-responsive NPs. Thus, we believe that the combination of *in situ* and *ex situ* methods for observing the crystallization of solid-state nanomaterials presented here will allow us to purposely direct the preparation of NP solids toward the desired properties.

## Experimental Section

### Organic Synthesis of L Compound

The L ligand was synthesized following a previously developed route.^51^

### Nanoparticles Synthesis

Spherical gold nanoparticles coated with dodecanethiol (Au@DDT) were prepared according to a modified literature method.^52^ Dodecylamine (3 g) was dissolved in cyclohexane (100 mL), then 12 mL of aqueous formaldehyde solution (37%) was added. After the mixture was vigorously stirred for 10 min, the organic phase was separated out and washed twice with water (2 × 30 mL). Next, an aqueous solution of tetrachloroauric acid (0.08 g of HAuCl_4_ in 20 mL of water) was added under vigorous stirring. After being further stirred for 40 min, the cyclohexane phase was separated out by centrifugation. Next, an excess of dodecanethiol (2 mL) was added, and the reaction mixture was stirred overnight. The formed precipitate was centrifuged (10 min, 6000 rpm) and rejected, and then 150 mL of ethanol was added to the solution. The precipitate was centrifuged (10 min, 6000 rpm), collected, and dissolved in a small amount of cyclohexane (10 mL). The precipitation procedure was repeated two more times. After that, the obtained cyclohexane solution was again centrifuged (10 min, 8000 rpm), in order to get rid of insoluble aggregates. To get fractions of NPs with a rather narrow and small size distribution, a fractionation process was performed. A small amount of ethanol was added to the cyclohexane NPs solution until turbidity appeared. The precipitate was centrifuged (10 min, 6000 rpm) and dissolved in a small amount of cyclohexane, and to the remaining supernatant, a further portion of ethanol was added. The process described above was repeated three additional times, yielding fractions containing smaller and smaller NPs.

Details about the obtained NPs with histograms of their size distribution, as derived from the TEM image, are available in the Supporting Information (Figure S1).

### Ligand Exchange Reaction on Nanoparticles

We introduced a promesogenic ligand L into NPs surface by the ligand-exchange reaction.^53^ To 10 mg of Au@DDT dissolved in 2 mL of cyclohexane, 25 mg of the L compound dissolved in 5 mL of dichloromethane was added. The reaction mixture was stirred slowly for 12 h. Then, the solvents were evaporated under reduced pressure, and the NPs were dissolved in 3 mL of toluene. Next, the NPs were precipitated with the addition of 10 mL of ethanol and centrifuged (10 min, 6000 rpm). The supernatant, containing unbound thiols, was rejected, and the precipitate was dissolved in 3 mL of toluene. This washing procedure was repeated a few times, until no unbound ligand was present, as determined with thin-layer chromatography. After the last precipitation, the NPs were dissolved in 5 mL of dichloromethane.

### Thermogravimetric Analysis

TGA analysis was performed with a TA Q50 V20.13 (TA Instruments) analyzer. The measurements were carried out in the 100–900 °C range with a 10 °C/min heating rate in a nitrogen atmosphere.

We observed two distinct steps of this weight loss (Figure S3a). The first one took place below 260 °C and corresponds to the removal of the alkyl coverage of nanoparticles (dodecanethiol molecules). A wider loss at a temperature above 260 °C is due to the removal of promesogenic ligand molecules. To recalculate the obtained data, we first calculated the mass of a single nanoparticle, using the average diameter derived from SAXRD and TEM and the bulk density of metals. The mass of organic compounds (*m*_org_) removed from a single nanoparticle was calculated using the percentage of mass left after the analysis (%*m*_left_), percentage of mass loss for a given mass loss (%*m*_loss_, separately for mass loss below and above 260 °C), the mass of a single Au NP core (*m*_Au_, calculated using bulk Au density): *m*_org_ = *m*_Au_/%*m*_left_ × %*m*_loss_. To determine the number of ligands per nanoparticle, the *m*_org_ for the given mass loss must be divided by the mass of a single molecule of ligand responsible for the mass loss (dodecanethiol for the mass loss below 260 °C and L for the mass loss above 260 °C).

### NMR Analysis of Hybrid Material

Twenty milligrams of Au@L nanoparticles, directly after purification, were used. Dichloromethane was evaporated under reduced pressure, and then NPs were dissolved in 1 mL of CDCl_3_. NMR spectra revealed the presence of broad signals in positions characteristic of the L ligand, which also allowed us to conclude that unbound ligands were removed in the workup process.^60^

### SAXRD Measurements

The small-angle X-ray diffraction (SAXRD) measurements were performed with a Bruker Nanostar system (Cu Kα radiation, parallel beam formed by cross-coupled Goebel mirrors and 3-pinhole collimation system, area detector VANTEC 2000). The temperature of the sample was controlled with a precision of 0.1 °C. Samples were prepared as a thin film on a Kapton tape substrate. For all samples temperature-dependent measurements were performed in the same manner—data were collected every 5 °C for 60 s. A quasi-monodomain sample was prepared by a mechanical shearing at elevated temperatures (10 °C below the phase transition point) on a heating table.

Fitting of the experimental diffractograms and simulation of the patterns were done using Topas 3 software (Bruker). Each procedure started with choosing the most probable symmetry of the lattice. Then, the unit cell parameters, intensities of the (Pseudo-Voigt) signals, and (1/*x*) background intensity were considered as independently adjustable parameters.

### Transmission Electron Microscopy

TEM measurements were performed using a JEM-1011 (JEOL) microscope equipped with a model EDS INCA (Oxford) analyzer (Mossakowski Medical Research Centre Polish Academy of Sciences, Warsaw). For TEM imaging materials, a diluted NP solution was dropcasted into TEM grids and then thermally treated on a heating table.

To determine the average monodomain area, ImageJ software was used. In the case of each cooling rate, three various TEM images were chosen, and an area of 100 monodomains was calculated. These results were used to determine the average monodomain area for all cooling rates.

HAADF-STEM imaging and tomography were performed using a ThermoFischer Scientific Osiris electron microscope operated at 200 kV and an aberration-corrected ThermoFischer Scientific Titan Cubed electron microscope, operated at 300 kV (Electron Microscopy for Materials Research, Antwerp).

The thermal treatment began with heating the samples from room temperature to 120 °C, with a heating rate of 4 °C/min, followed by cooling them again to room temperature. Although the heating rate was the same in both experiments, the cooling rate was different, 3 and 0.5 °C/min for the first and second experiment, respectively. Moreover, in the first experiment, the sample was treated thermally *ex situ* in an oven (Carbolite CWF 1200) with activated carbon, and in the second investigation, the treatment was performed *in situ* using a DENSsolutions Wildfire tomography heating sample holder.

In the *ex situ* experiment (Figure 4e), the HAADF-STEM tomography tilt series was acquired from −51° to +77° and a tilt increment of 2° using a Fischione model 2020 single-tilt tomography holder. A camera length of 115 mm was used.

In the *in situ* experiment (Figure 3b−g) , the HAADF-STEM tomography tilt series was acquired using a DENSsolutions Wildfire heating sample holder optimized for electron tomography. The first (Au@L_d__rop_) and second (Au@L_0.5_) series were acquired within a ±70° range and a tilt increment of 3°, while a ±65° range and a tilt increment of 5° was used in the third series, to avoid the effect of carbon contamination. A camera length of 115 mm was used.

At the individual HAADF-STEM projection images of the tomography series (Figure 3h–m), we applied nonrigid registration methods in combination with a convolutional neural network (CNN) to eliminate different distortions.^61,62^ This protocol uses multiple successive images as an input; in this case, 10 frames of 2k × 2k with a scan time of 0.5 s for each frame consecutively. Undistorted images were aligned using a phase correlation, which was also used to determine the shift and the angle of the rotation axis. A 3D reconstruction of the aligned series was performed by iterating between 25 simultaneous iterative reconstruction technique cycles and the application of constraints in the real and Fourier space. After a bandwidth limit was applied to the FFT, the result was transformed to real space, and a threshold was applied to the intensity of the 3D volume. The 3D-FFT was segmented, and a mask was obtained, from where the inverse fast Fourier transform was obtained, allowing the retrieval of the unit cell.^63,64^

### UV–Vis Spectroscopy

The UV–vis spectra were acquired using a Cary 5000 spectrometer (Agilent). The aggregates were recorded on a glass substrate in a transmission mode.

### Optical Simulation Methods

To simulate the optical properties of plasmonic superlattices, we applied a multiple scattering code for metallic NPs,^65^ with periodic boundary conditions in the lateral direction. It eventually solves Maxwell’s equations for such a specific geometry. Five unit cells in a longitudinal direction were considered, assuming a perfectly periodic arrangement of spherical Au NPs. In the lateral direction, the number of particles is infinite.

For the simulation of finite sample sizes, we used an implementation of the Generalized Multiparticle Mie Method.^66^ The scattering of up to ∼10 000 NPs was calculated, with varying degrees of disorder within the domains.

### AFM Measurements

AFM imaging was realized using Agilent 5500 atomic force microscopy working in tapping mode. Soft tapping mode cantilever All-In-One Al (Budget Sensors) probe C was used, with a typical force constant of 7.4 N/m and resonant frequency of ca. 150 kHz. The typical scan frequency was 0.5 Hz. These measurements were performed in the Wielkopolska Centre for Advanced Technology, Poznań.

## Supporting Information Available

Additional figures: characterization of NPs, an analysis of the surface coverage of Au@L NPs (NMR and TGA analysis), investigation of the Au@L material structure depending on the number of the thermal annealing cycles, additional TEM images used to calculate the average domain area, analysis of AFM measurements. Additional tables summarize liquid-crystalline phases formed by Au@L NPs and plasmonic band maxima positions of the analyzed samples (PDF)
(MP4)
(MP4)
(MP4)
(MP4)
(MP4)
(MP4)
