# Supplementary material for: Understanding and Controlling the Crystallization Process in Reconfigurable Plasmonic Superlattices
Source: ACS Nano. 2021 Feb 23;15(3):4916–26. doi: 10.1021/acsnano.0c09746 (PMC8028333; doi:10.1021/acsnano.0c09746)
Supplement: Supplementary file 1 — nn0c09746_si_001.pdf [file nn0c09746_si_001.pdf]

Supporting Information

for

Understanding and Controlling the Crystallization Process in Reconfigurable Plasmonic Superlattices

Maciej Bagiński, Adrián Pedrazo-Tardajos, Thomas Altantzis, Martyna Tupikowska, Andreas Vetter, Ewelina Tomczyk, Radius N.S. Suryadharma, Mateusz Pawlak, Aneta Andruszkiewicz, Ewa Górecka, Damian Pociecha, Carsten Rockstuhl, Sara Bals, Wiktor Lewandowski

Table of contents

Supplementary Figures

Figure S1. Characterization of primary (Au@DDT) NPs.

Figure S2. UV-Vis spectra collected for Au@L dispersion in toluene.

Figure S3. Analysis of surface coverage of Au@L.

Figure S4. Structural characterization of Au@L.

Figure S5. Temperature evolution of Au@L SAXRD pattern during cooling.

Figure S6. Investigation of Au@L material structure depending on the number of thermal annealing cycles.

Figure S7. TEM images used to calculate average monodomains area for samples with various number of thermal annealing cycles.

Figure S8. AFM measurements of Au@L assemblies.

Figure S9. TEM images used to calculate average monodomains area for samples with various cooling rate.

Figure S10. Additional information for *in situ* investigation of the crystallization conditions effects on Au@L material structure shown in **Figure 3** in the main text.

Figure S11. HAADF-STEM analysis of Au@$L_{0.5}$.

Figure S12. Contraction of material under the electron beam irradiation.

Figure S13. Comparison of experimental and simulated extinction spectra for Au@L_{bcc} NPs.

Figure S14. TEM image revealing isotropic order of NPs at the edges of domains for Au@L_3.

Supplementary Tables

Table S1. Summary of liquid-crystalline phases formed by Au@L NPs.

Table S2. Summary of plasmonic band maxima positions of analyzed samples.

Supplementary Movies

Movie S1. Colored and grayscale visualization of the HAADF-STEM tomography corresponding to Au@L_{drop}, the corresponding 3D-FFT and the slices through the 3D reconstruction, of the general region shown in **Figure 3** panels **c** and **d** in the main text.

Movie S2. Colored and grayscale visualization of the HAADF-STEM tomography corresponding to Au@L_3, the corresponding 3D-FFT and the slices through the 3D reconstruction, as shown in **Figure 4e** in the main text.

Movie S3. Colored visualization of the HAADF-STEM tomography corresponding to Au@$L_{0.5}$, the corresponding 3D-FFT and the slices through the 3D reconstruction, of the general region shown in **Figure 3** panels **f** and **g** in the main text.

Movie S4. Grayscale visualization of the HAADF-STEM tomography corresponding to Au@$L_{0.5}$ from the single domain shown in **Figure 3** panels **h**, **j** and **l** in the main text. The 3D-FFT is superimposed with the original 3D reconstruction colored in green.

Movie S5. Grayscale and colored visualization of the HAADF-STEM tomography corresponding to a section of the tomography shown in Movie **S4**. The 3D-FFT is superimposed on both reconstructions colored in green.

Movie S6. HAADF-STEM time series, with a time frame of one second, from Au@$L_{0.5}$ showing the contraction of the domains under the electron beam as demonstrated in Figure **S12**.

(a) (b)

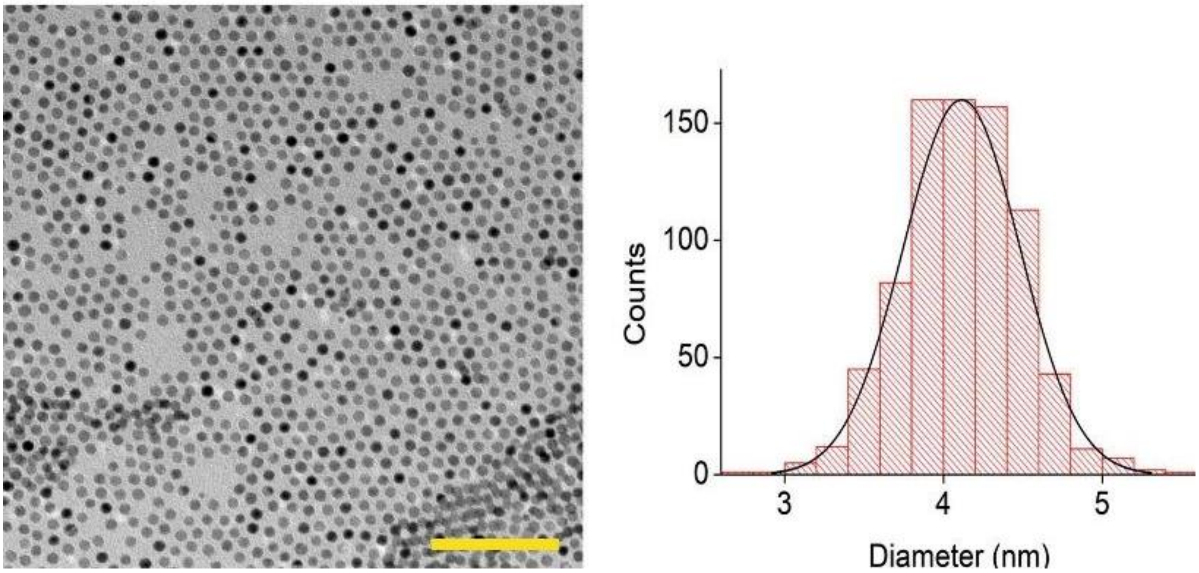

Figure S1. Characterization of primary (Au@DDT) NPs. (a) TEM image and (b) histogram of their size distribution. Scale bar in panel a is 50 nm.

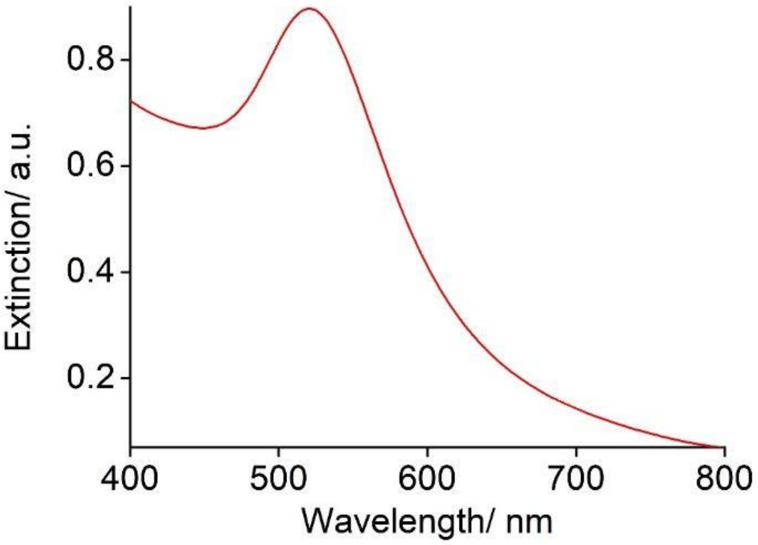

Figure S2. UV-Vis spectra collected for Au@L dispersion in toluene shows clear plasmonic band centered at ~520 nm.

(a)

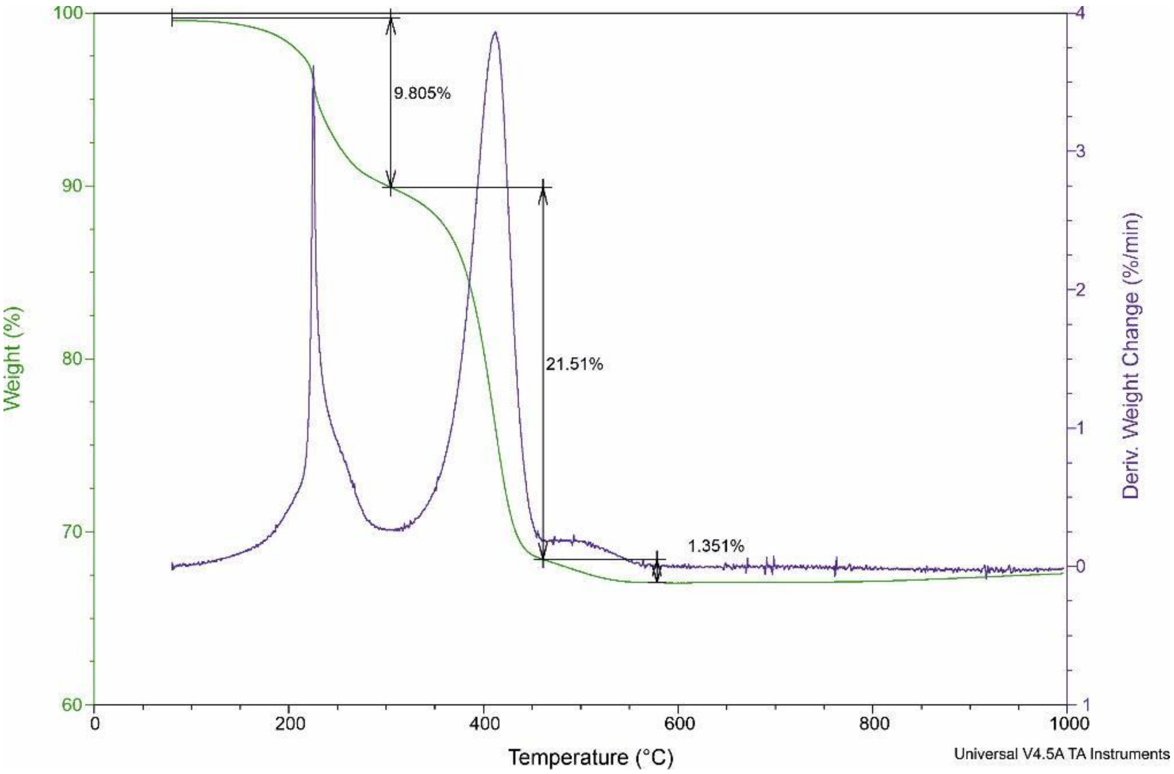
Weight (%)
Deriv. Weight Change (%/min)
Temperature (°C)
9.805%
21.51%
1.351%
Universal V4.5A TA Instruments
100
90
80
70
60
0
200
400
600
800
1000
4
3
2
1
0
1

(b)

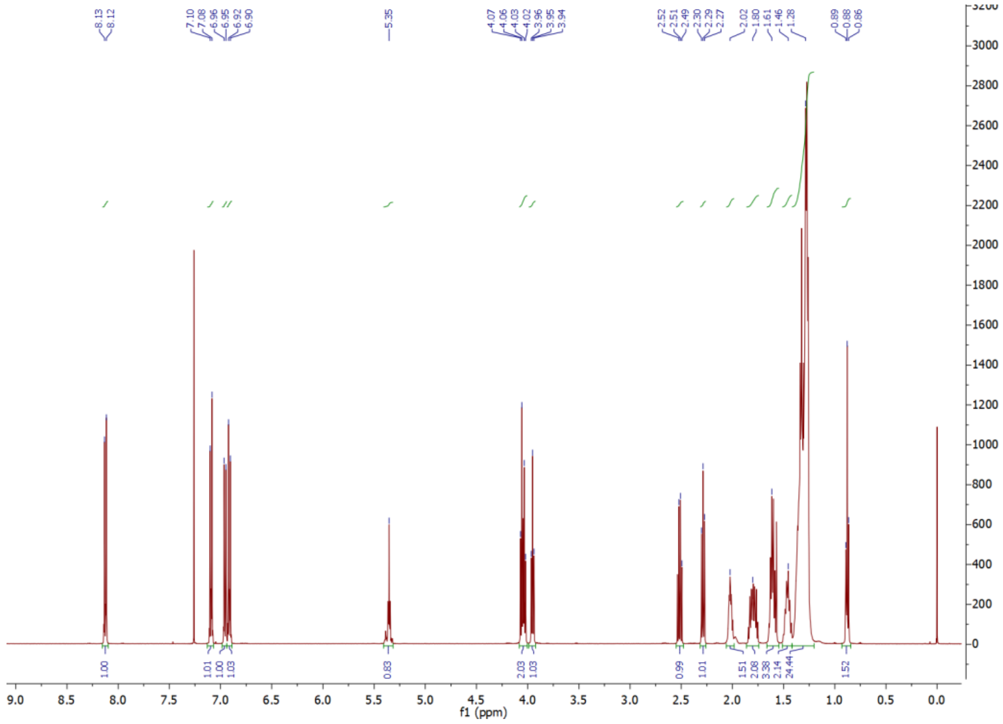
8.13
8.12
7.10
7.08
6.96
6.95
6.92
6.90
5.35
4.07
4.06
4.03
4.02
3.96
3.95
3.94
2.52
2.51
2.49
2.30
2.29
2.27
2.02
1.80
1.61
1.46
1.28
0.89
0.88
0.86
1.00
1.01
1.00
1.03
0.83
2.03
1.03
0.99
1.01
1.51
2.08
3.38
2.14
24.44
1.52
f1 (ppm)

(c)

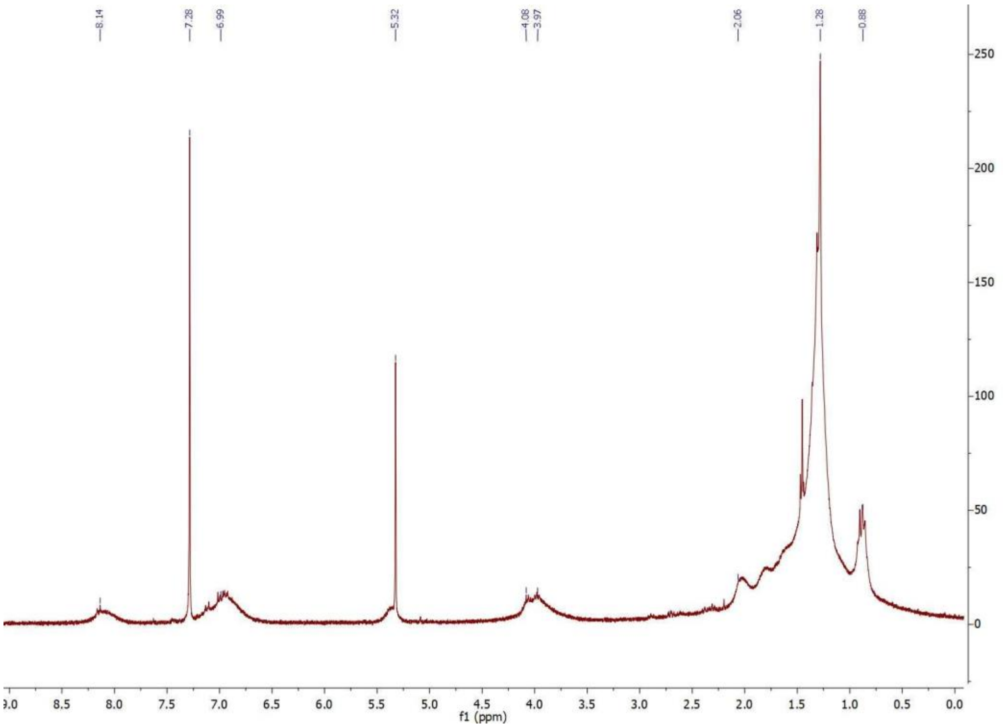

Figure S3. Analysis of surface coverage of Au@L. (a) TGA curve showing weight loss of the sample during heating (green line) and derivative of the weight loss (purple line); (b) 1HNMR spectra of L; (c) 1HNMR spectra of Au@L For Au@L NPs we can observe the same position of signals as for L, but they are broad. Moreover, NMR spectra also indicated the absence of free ligands based on the absence of –CH_2S moiety signal (signal located at 2.52 ppm). These observations confirm successful of ligand exchange reaction. Sharp signals located at 7.28 ppm and 5.32 ppm are from solvents: chloroform and dichloromethane, respectively.

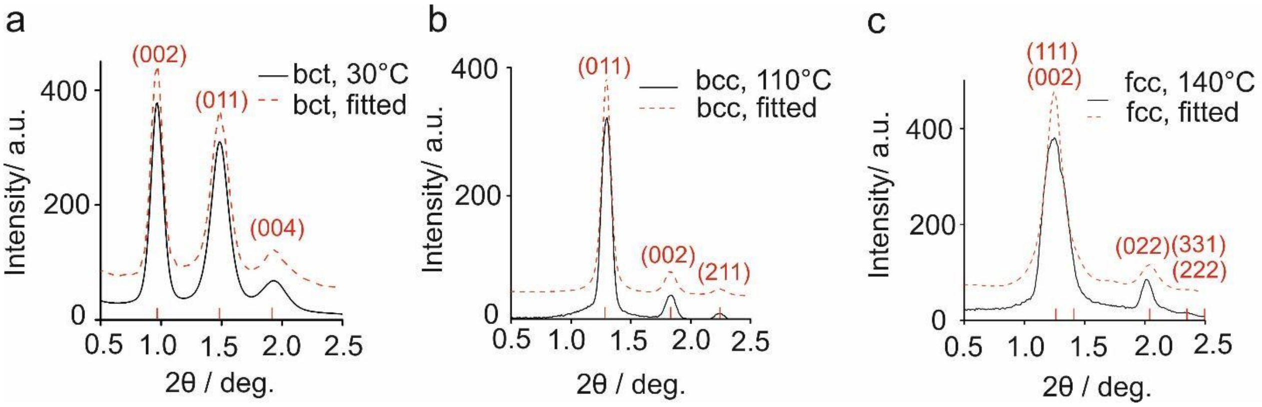

Figure S4. Structural characterization of Au@L. Comparison of modelled (red dotted line) and measured (black line) SAXRD profile for (a) lower (30 °C); (b) medium (110 °C) and (c) higher (140 °C) temperature phases.

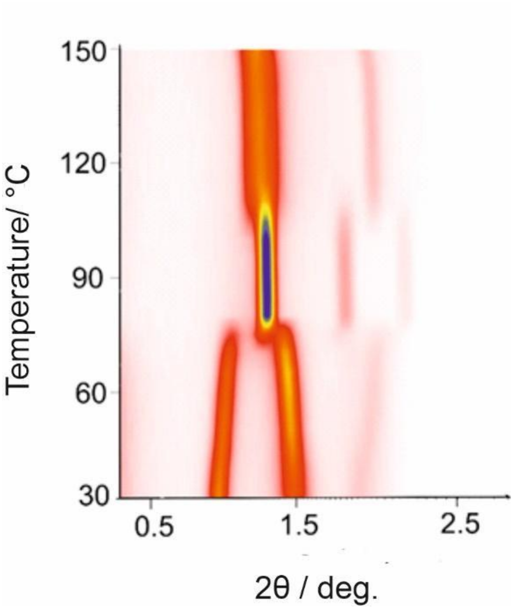

Figure S5. Temperature evolution of Au@L SAXRD pattern during cooling.

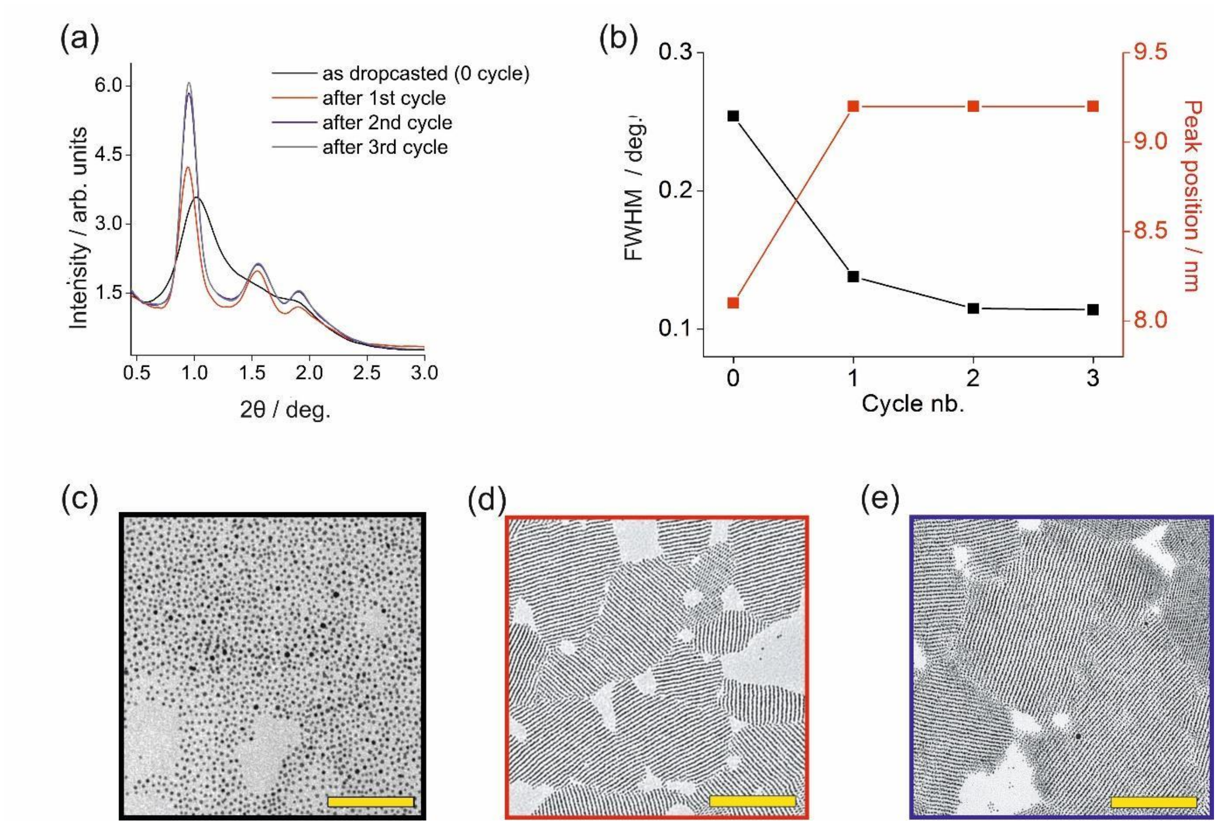

Figure S6. Investigation of Au@L material structure depending on the number of thermal annealing cycles. (a) Comparison of 1D diffractograms obtained for samples after 0-3 annealing cycles. (b) Comparison of position and full width at half maxima of the main XRD peak shown in panel a. (c-e) TEM images of samples: (c) dropcasted (without thermal treatment), (d) after the 1st annealing cycle and (e) after the 2nd annealing cycle. Colors of brackets around TEM images correspond to diffractograms shown in panel a. Scale bars: 100 nm in (c) and 200 nm in (d and e).

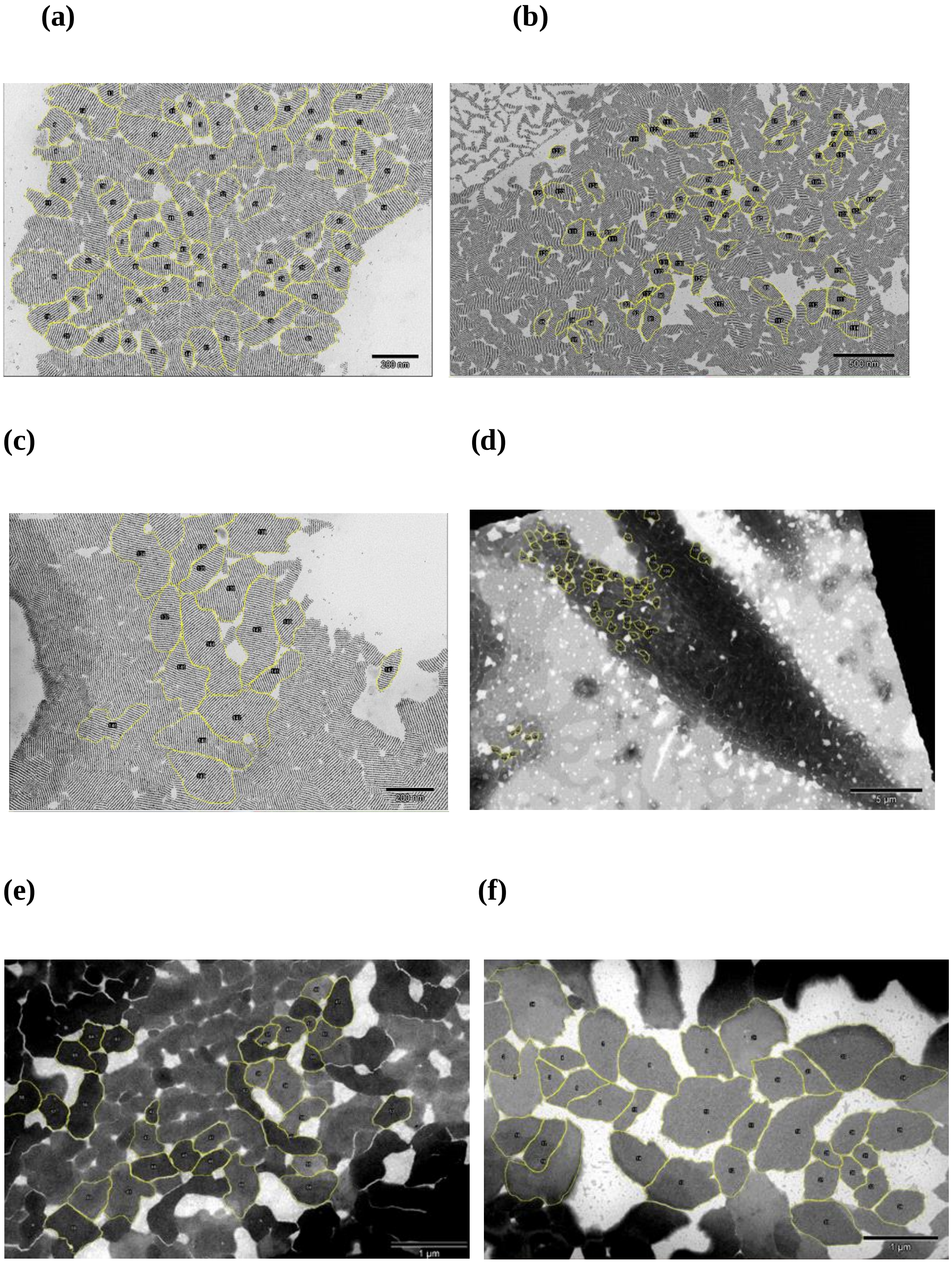

Figure S7. TEM images used to calculate average monodomains area for samples with various number of thermal annealing cycles: (a-c) after 1st cycle; (d-f) after 2nd cycle.

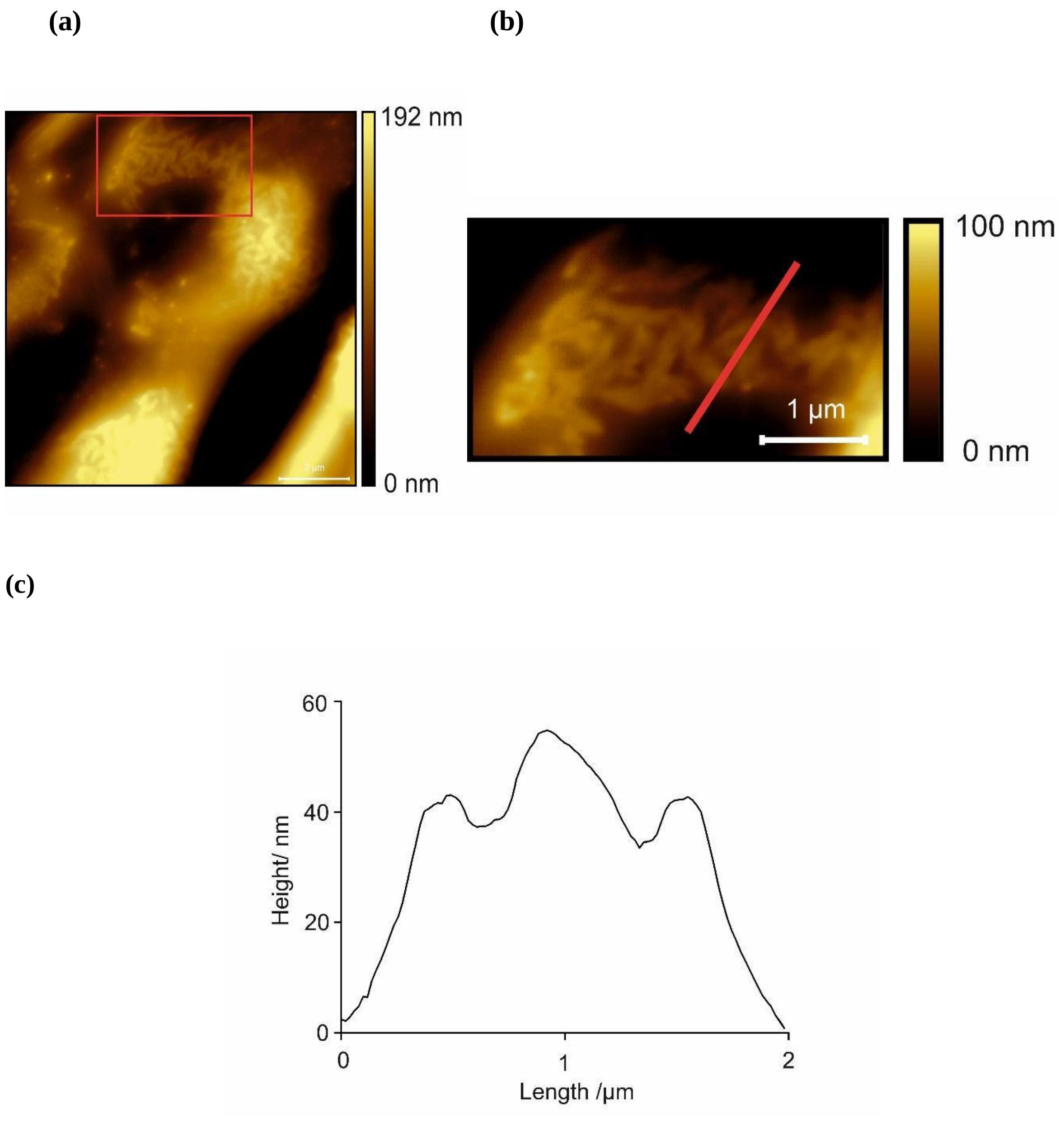

Figure S8. AFM measurements of Au@L assemblies. (a,b) AFM height images of a sample dropcasted onto a TEM grid; panel b shows a magnified area of the image shown in panel a, indicated by the red frame. (c) Height profile from the AFM image shown in panel b, taken along the red line.

(a)

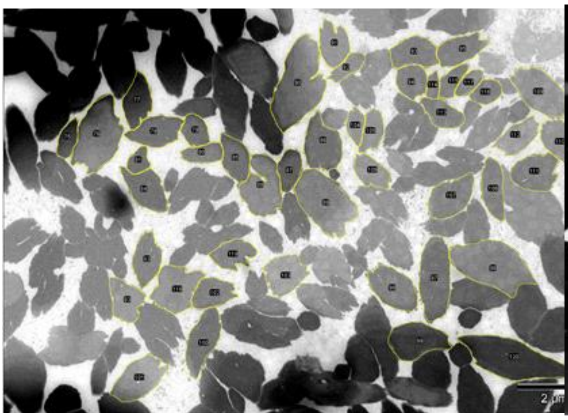

(b)

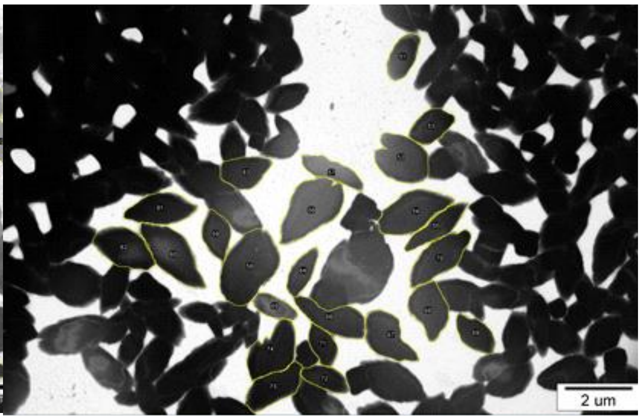

(c)

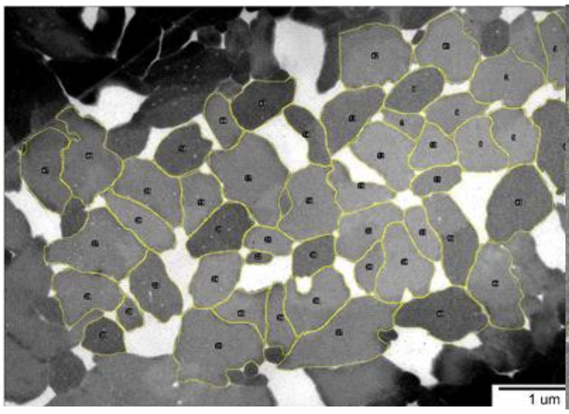

(d)

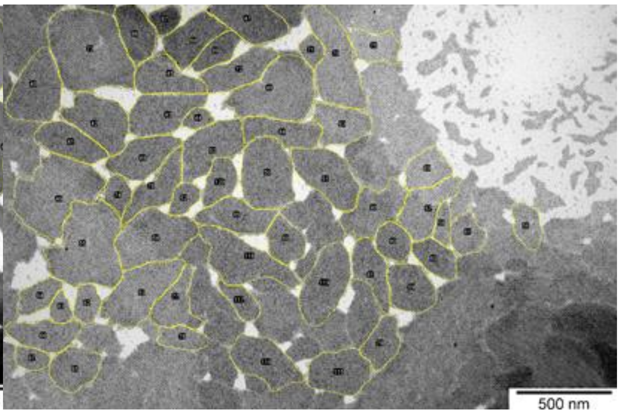

(e)

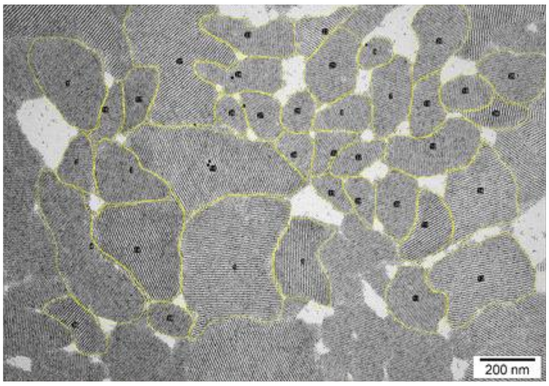

(f)

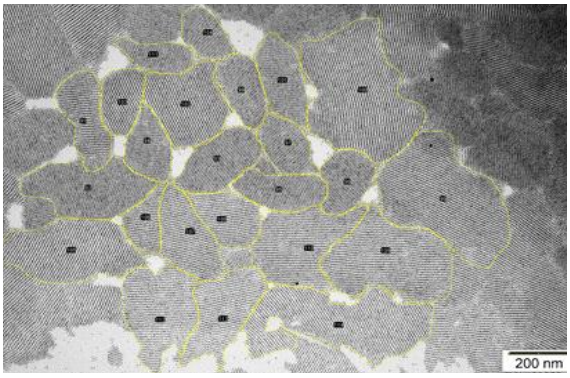

(g)

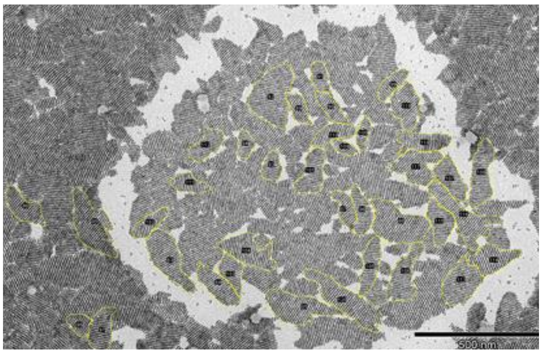

(h)

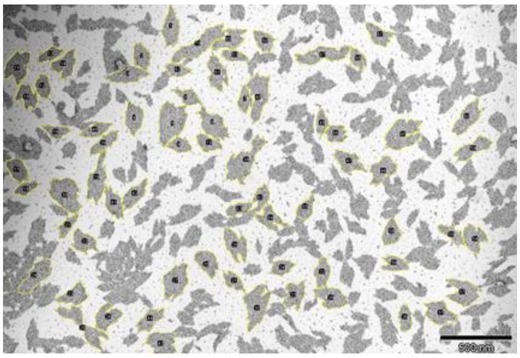

(i)

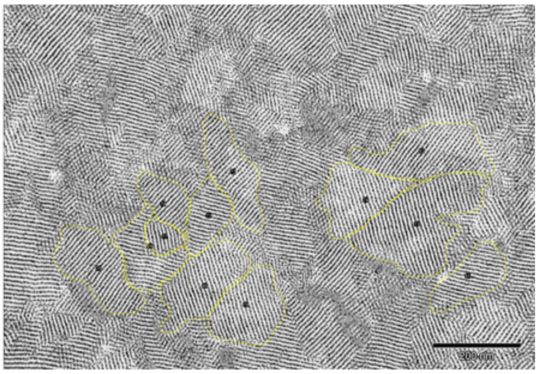

(j)

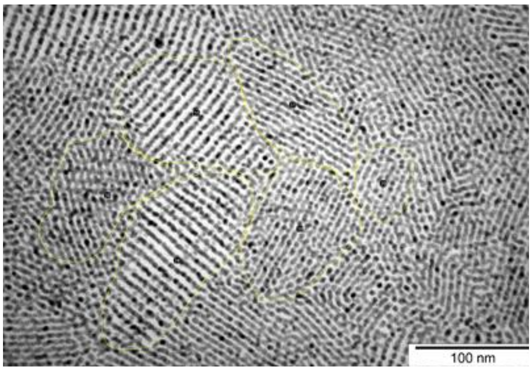

(k)

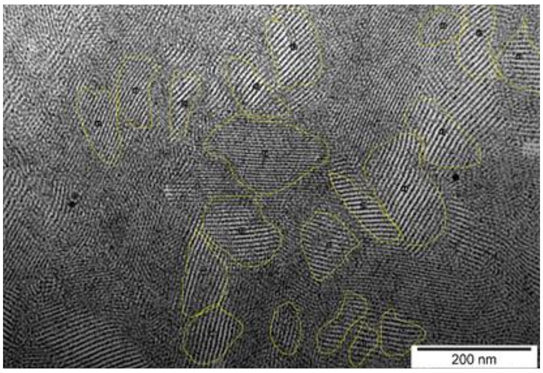

(l)

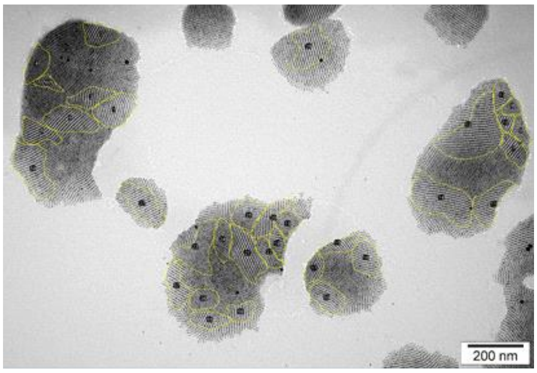

Figure S9. TEM images used to calculate average monodomains area for samples with various cooling rate: (a-c) 0.5 °C/min; (d-f) 8 °C/min; (g-i) 15 °C/min; (j-l) 30°C/min.

(a)

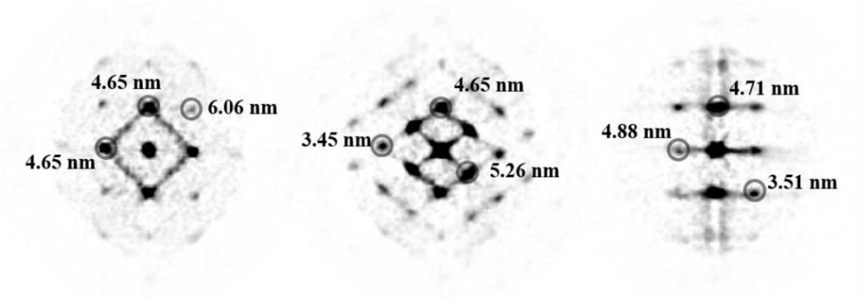

(b)

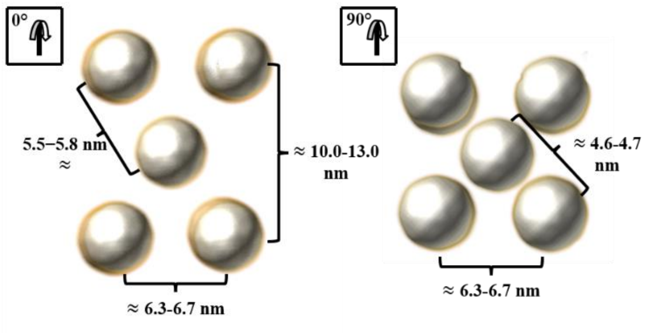

Figure S10. Additional information for *in situ* investigation of the crystallization conditions effects on Au@L material structure shown in **Figure 3** in the main text. (a) Simplified FFTs corresponding to those shown in **Figure 3i, k and m**, respectively (b) Real space dimensions calculated based on the IFFT analysis, in good correspondence with the values shown in panel (a).

(a)

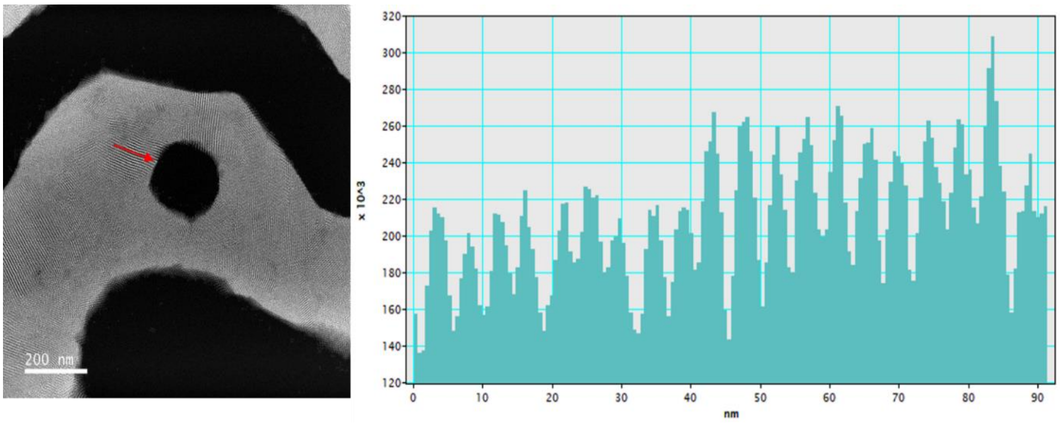

(b)

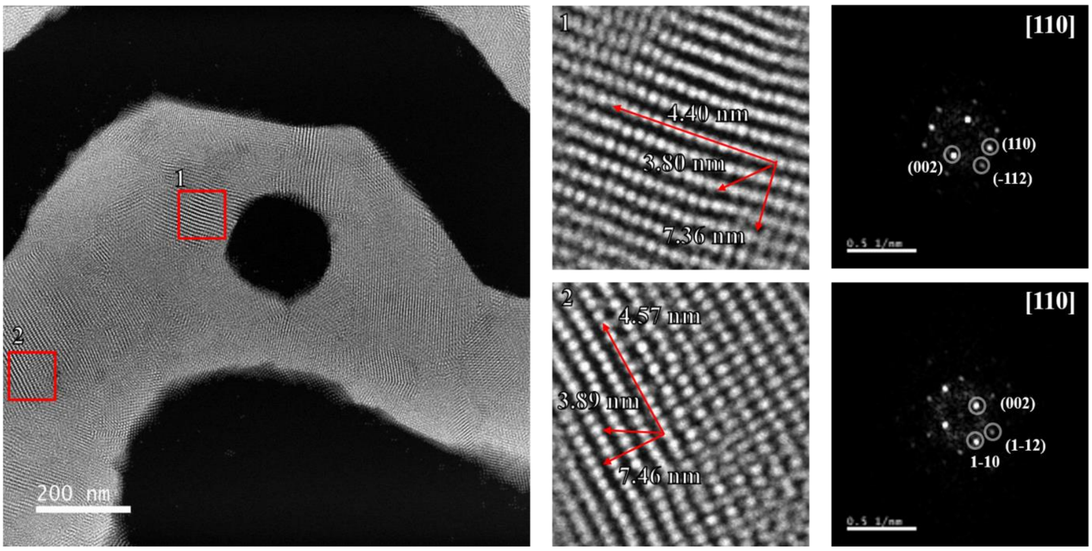

Figure S11. HAADF-STEM analysis of Au@$L_{0.5}$. (a) HAADF-STEM image of a polydomain structure (on the left). Grayscale measurements showing the interparticle distances (center-to-center) along the red line shown in the image, which was used to determine the mean unit cell dimensions (on the right). (b) Analysis of the unit cell dimensions of Au@$L_{0.5}$ in two regions highlighted with red squares. Interparticle distances along crystallographic directions [002][110][112] are highlighted, FFT images corresponding to highlighted regions (on the right) indicating a [110] zone axis for both of them.

(a) **(b)**

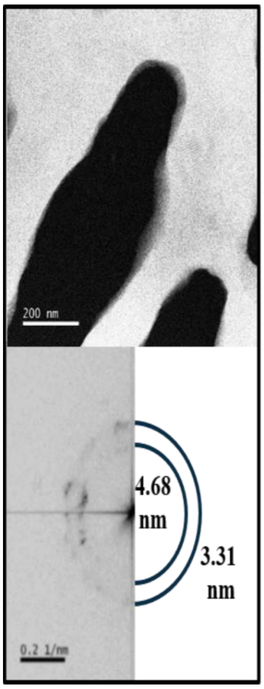

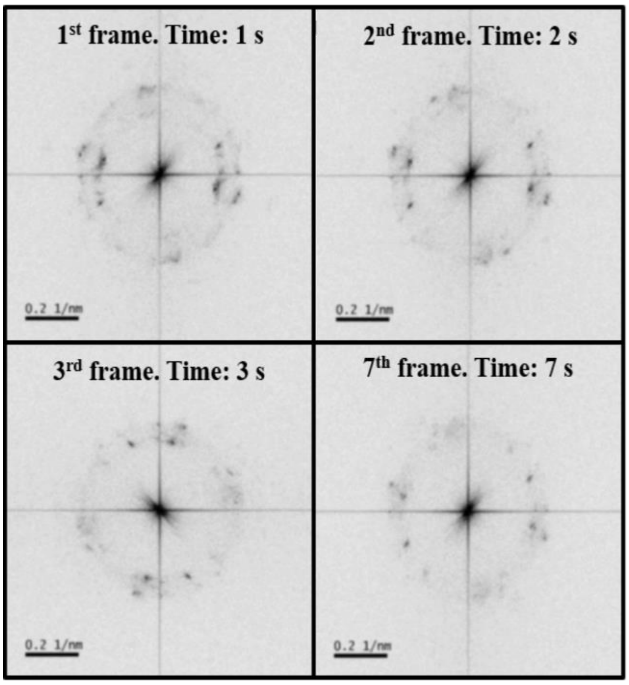

(c)

Total time (s)	Distance (nm) measured from the ring pattern	
1	1st ring 4.68 nm	2nd ring 3.31 nm
2	1st ring 4.04 nm	2nd ring 3.29 nm
3	1st ring 3.97 nm	2nd ring 3.29 nm
7	1st ring 3.90 nm	2nd ring 3.29 nm

Figure S12. Contraction of material under the electron beam irradiation: a) Frame from the HAADF-STEM time series from Au@$L_{0.5}$ and the corresponding FFT pattern revealing the d-spacing. b) Different frames from the **Movie S6** showing contraction of material under the electron beam irradiation. c) Table with the estimation of the d-spacing in several frames. The structure loses around 20% of its size within 7 seconds, after which it becomes stable.

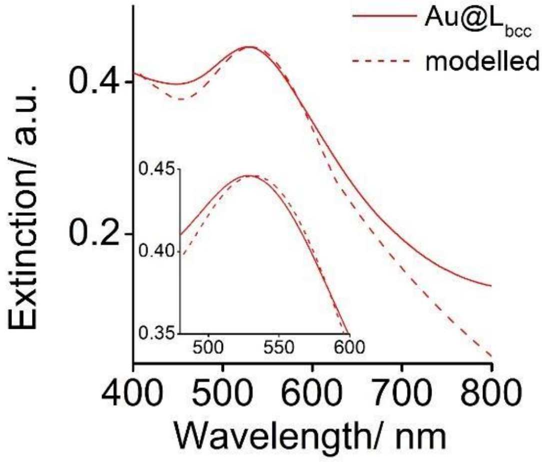

Figure S13. Comparison of experimental and simulated extinction spectra for Au@L$_{bcc}$ NPs.

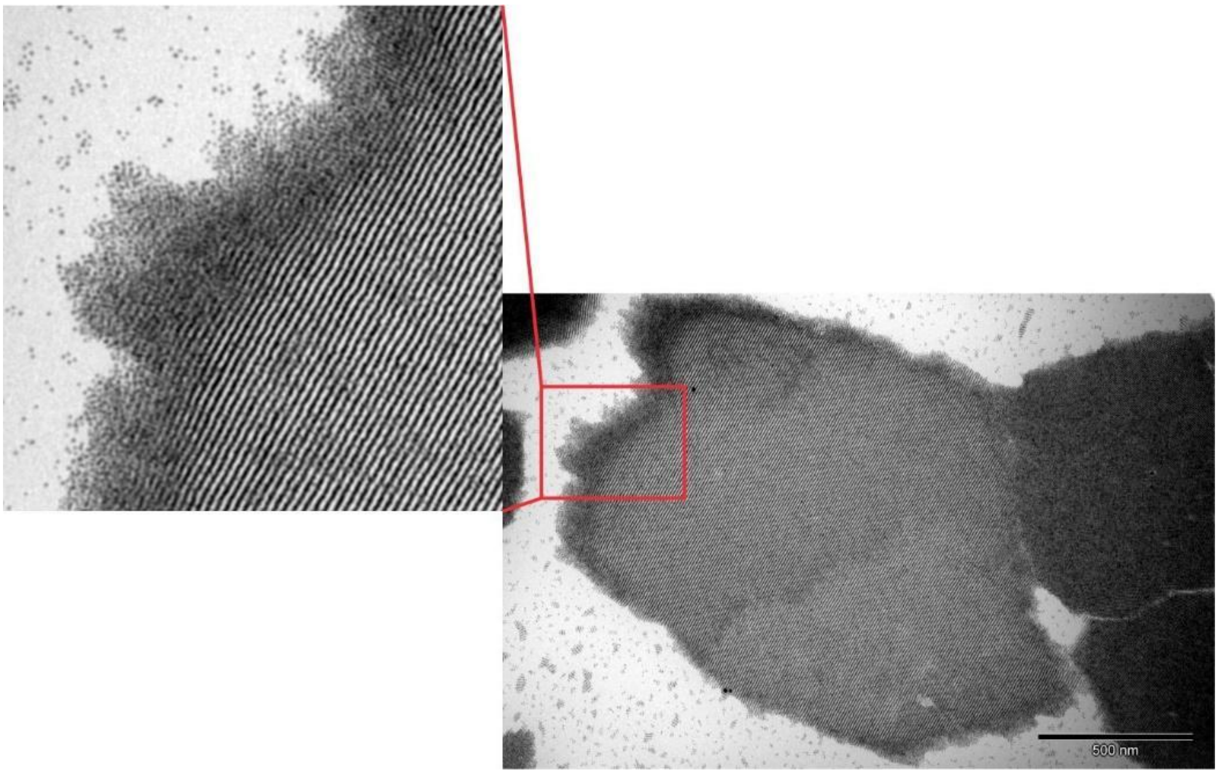

Figure S14. TEM image revealing isotropic order of NPs at the edges of domains for Au@L$_3$.

Table S1. Summary of liquid-crystalline phases formed by Au@L NPs.

**Temperature (˚C) / phase**	**hkl plane index**	**d (Å) experimental**	**d (Å) fitted**
30<br>bct phase	(002)	91.6	91.5
	(011)	59.5	59.7
	(004)	45.6	45.7
110<br>bcc phase	(011)	68.3	68.5
	(002)	48.3	48.4
	(211)	39.4	39.5
140<br>fcc phse	(111)<br>(002)	69.8 (broad)	70.3<br>62.4
	(022)	43.4	43.2
	(331)<br>(222)	37.6 (broad)	36.9<br>35.4

Table S2. Summary of plasmonic band maxima positions of analyzed samples.

Sample	Plasmonic band maxima position (nm)/experiment	Plasmonic band maxima position (nm)/modelled
**Au@L dispersion in toluene**	520	-
**Au@L_{drop}**	535	540
**Au@L_3**	551	548
**Au@$L_{0.5}$**	554	551
**Au@L_{bcc}**	530	532
